# Self-healing neural networks via modular patch layers for diverse structural and adversarial damages

**DOI:** 10.1038/s41598-026-57677-x

**Published:** 2026-06-22

**Authors:** B. Santhosh Reddy, S. Deepa Nivethika, B. Vara Anjan, P. Mahidhar Reddy, M. SenthilPandian, R. Roobankumar

**Affiliations:** 1 School of Computer Science and Engineering, Vellore Institute of Technology, Chennai, 600127 Tamil Nadu India; 2 School of Civil Engineering, Vellore Institute of Technology, Chennai, 600127 Tamil Nadu India; 3 Department of Civil Engineering, PERI Institute of Technology, Chennai, 600048 Tamil Nadu India

**Keywords:** Neural networks, Self-healing, Structural damage, Adversarial, Patch-based repair, deep learning, activation deviation, FGSM, PGD, Keras, Streamlit, Engineering, Mathematics and computing

## Abstract

The growing use of modern deep neural networks (DNNs) in safety-critical and continuously operating systems exposes them to safety-centric concerns and raises the usual challenge of maintaining their continued operation. Internal structural flaws or adversarial attacks may lead to performance degradation that may severely affect reliability. This paper presents a Self-Healing Neural Network (SHNN) model which can enhance recovery in post-deployment by automatically identifying, localizing, and repairing damaged components of networks with lightweight and modular patch layers. SHNN isolates faults without requiring full retraining or global fine-tuning by performing activation discrepancy analysis at each layer and retraining the afflicted layer selectively, which causes little computational and memory overhead. Experiments of MNIST, Fashion-MNIST, Sign Language MNIST, and CIFAR-10 indicate SHNN restores performance after structural damage close to baseline (97.33% and 97.22% vs. 97.76% test accuracy of the zeroed and randomised weights) and restores a lost accuracy of 74–84% in FGSM and PGD adversarial attacks. Post-healing activation deviation scores decrease significantly, confirming effective fault localization as the reduced value confirms. The framework offers a self-repair mechanism based on modules, interpretable, and dataset adaptive. with a live Streamlit interface, and allows building resilient and fault-tolerant AI systems to operate in high-reliability environments.

## Introduction

Deep neural networks (DNNs) have shown good performance in many fields, such as in computer vision, natural language processing, healthcare, and intelligent sensing systems. However, their reliability in practice remains a great issue. There are two major types of threats that are faced in operational contexts of these models that include internal structural degradation and adversarial intervention. Hardware may become old, the memory is corrupted, the pruning occurs by accident, or the quantization effects may occur, leading to structural damage. These usually occur when no one notices and disrupt internal calculations thus leading to a significant performance decrease. In addition to the traditional approaches such as CNN^1^ Constraint-based and optimization-driven repair models, such as NNRepair^2^, QNNRepair^3^, CARE^4^, Closed-Loop Self-Healing^5^, or AIREPAIR^6^ have demonstrated that this form of degradation can be reduced, but it tends to increase the effort of the retraining process. Retraining is infeasible in a number of real-time or resource-constrained conditions, and thus, it is highly recommended that, in this context, alternative, lightweight and efficient self-repair mechanisms be developed^7^. Meanwhile, DNNs are highly susceptible to adversarial attacks, when carefully crafted and imperceptible images result in incorrect predictions. Gradient-based methods such as FGSM and PGD are always known to follow attack vulnerabilities in learnt decision boundaries, as demonstrated by PatchZero^8^ and DIFFender^9^. PATCHOUT^10^ demonstrated that semantic consistency analysis can be applied to locate adversarial patches in a given area. Patch detection methods grounded on explainability have been presented in AdvPatchXAI^11^. Segment and Complete^12^ demonstrated that adversarial patches can considerably complicate the operation of object detection algorithms and suggested that semantic segmentation can be used to resolve the issue. PAD^13^ indicated that the patch-agnostic suppression strategies might render it more difficult to be influenced by an enemy though you may not be aware of the shape and size of the patch. In practice, diffusion-based defenses have been demonstrated to be more robust to adversarial examples even in real world environments, with no constraints, with traditional UAV-specific defenses demonstrated to be more vulnerable in systems that recognize an object in flight^14^. RFENet^15^ enhanced the resilience of feature extractors used in aerial segmentation by stabilizing them to patch attacks. The combination of these researches demonstrates the extent and severity of hostile patch vulnerabilities.

### Structural damage in neural networks

There is a structural degradation of deployed neural networks; this is a persistent and poorly studied issue. Radiation bit flips and changes in voltage as well as long-term use might damage stored weights and activations slowly. Even minor modifications can propagate through layers as demonstrated by NNRepair^2^, whereas quantized networks are found to be more susceptible to such modifications as demonstrated by QNNRepair^3^. Causal modeling was used to identify the primary cause of failures of networks by CARE^4^ and broken inference pipelines could be repaired using feedback control theory by Closed-Loop Self-Healing^5^. A neuron pruning and reparameterization based automated method to repair failed networks was proposed by AIREPAIR^6^. The concept-based repair can be found in Semantic-Based Repair^7^, which established concept-based rules to ensure the consistency of classes. Most of these systems are however based on the repeated correction or part retraining which in a situation where resources are scarce and time is the order of the day may not augur well. An effective and label-free deep learning model that incorporates a batch-normalized domain adaptation and explainability to detect masonry cracks precisely in new environments without requiring new annotations^16^. These issues are particularly severe in edge and embedded systems, which are more susceptible to them due to the scarcity of hardware capabilities and extended work periods. The use of neural networks in places where they must be reliable and always work is demonstrated by Smart infrastructure platforms^17^, networking testbeds^18^, large-scale smart automation systems^19^, THz wireless communication applications^20^, and biomedical AI systems^21^.

### Adversarial attacks and patch-based threats

One of the largest dangers of deep learning systems is adversarial attacks. PatchZero^8^ demonstrated that adversarial patches can have a significant impact on classification decisions, whereas DIFFender^9^ demonstrated that diffusion-based generative modeling could reduce the impact of patches. PATCHOUT^10^ was based on semantic consistency to discover adversarial interference in a particular region, whereas AdvPatchXAI^11^ simplified the process of understanding what occurs during adversarial detection. Patch attacks in object recognition Segment-and-Complete^12^ identified semantic patches by a combination of inpainting and semantic segmentation. Patch-agnostic gradient suppression layers were developed by PAD^13^ and they allow you to be strong without any assumptions. Diffusion-based defenses applied in the real world^22^ made systems more resilient in wild and natural environments, although UAV-based defenses^14^ resolved issues with aerial detection systems. RFENet^15^ made aerial segmentation even more stable by making feature extraction more stable when there are hostile changes. Even with these improvements, most current defenses work at the input preprocessing level and don’t immediately fix harm to internal representations. This makes them less effective in the long run and less reliable for recovery.

### Motivation for a self-healing paradigm

As deep learning models are used more and more in systems that are important for safety and missions, resilience, adaptability, and fault tolerance have become more important. Closed-loop repair designs^5^ and semantic-guided repair techniques^7^ have shown that internal correction mechanisms greatly improve long-term stability. Many current solutions, on the other hand, depend on retraining, constraint optimization, or repeated corrective cycles, which add delay and make the computer work harder. This encourages the use of self-healing neural paradigms, where models find, locate, and fix problems on their own utilizing lightweight modular methods, which keeps operations running smoothly and reliably^2–4,6^.

### Self-Healing Neural Network Framework (SHNN)

This paper offers the Self-Healing Neural Network (SHNN) structure to address the issues of internal degeneration and external intervention. SHNN is a single system design that is a combination of continuous self-monitoring, layer-based damage localization and recovery designs. The patch-wise modular repair system of SHNN draws its inspiration on the mechanism of self-healing of biological tissue. It applies a small auxiliary neural patch, which is only trained to simulate the damaged layer and the other network elements remain fixed. Layer-wise deviations are detected using Mean Absolute Error (MAE) between activations. In all experiments, the damaged layer is selected strictly as the argmax of MAE without additional thresholds. and thus it becomes possible to detect the flaws with a high accuracy. A different neural patch is then trained on clean samples in structural damage and a combination of clean and adversarial samples in the event of an attack after the damage has been detected. This enables the ability to redefine functionality very accurately. This plan is complementary to previous models of repair that were constraint based^2^, quantization based recovery models^3^, causal intervention models^4^, and adaption based frameworks that were feedback based^5^.

Structural decay, adversarial interference, and recovery behavior may also be viewed in real time in the integrated Streamlit-based graphical interface. This renders it more applicable to education, diagnostics, and deployment settings founded on wise infrastructure platform^17^, industrial automation testbeds^19^, and embedded intelligence platforms^21^.

### Relationship to prior work

Earlier studies have studied numerous techniques of improving brain resilience, including constraint-based repair^2,1^, quantized model recovery^3^, causal intervention schemes^4^, automated repair pipelines^6^, feedback-driven control systems^5^, and semantic-based correction models^7^. On the second hand, adversarial defenses have been advanced to gradient-based detection^8^, diffusion-based purification^9^, semantic consistency^10^, explainability-based detection^11^, segmentation-based restoration^12^, patch-agnostic suppression^13^, diffusion sampling optimization^22^, UAV defense^14^, and robust feature extraction^15^.

SHNN mends in a layer wise manner not like other approaches that simply use input space purification or global retraining. SHNN allows repairing structural failures including attacks by detecting the damage via activation mismatch and inserting lightweight patch modules. This does not affect the integrity of the still intact parts of the network.Compared to previously used repairing techniques that would require weight updating or solving the constraint, SHNN will freeze base weights and create modular compensation.

## Methodology

This subsection gives the architecture design, damage detection simulation plan and approach of healing combined with Self-Healing Neural Network (SHNN) system. It is a process that localizes and deploys structural and adversarial flaws in a neural network by modular patch layers. Everything is developed in Python with Keras to model and Streamlit to generate a user interface.

### Base model architecture

**Fig. 1:**
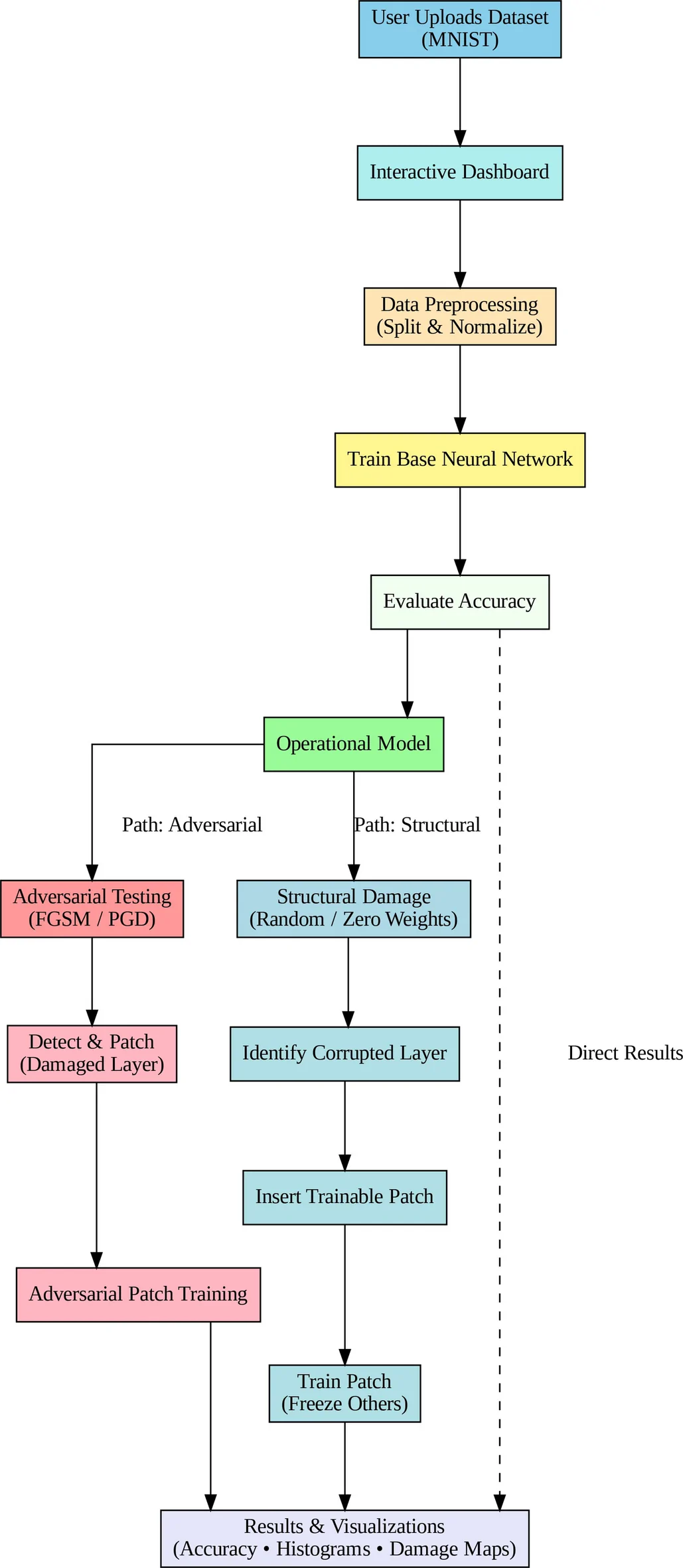
Neural Network (SHNN) system architecture.

**Fig. 2:**
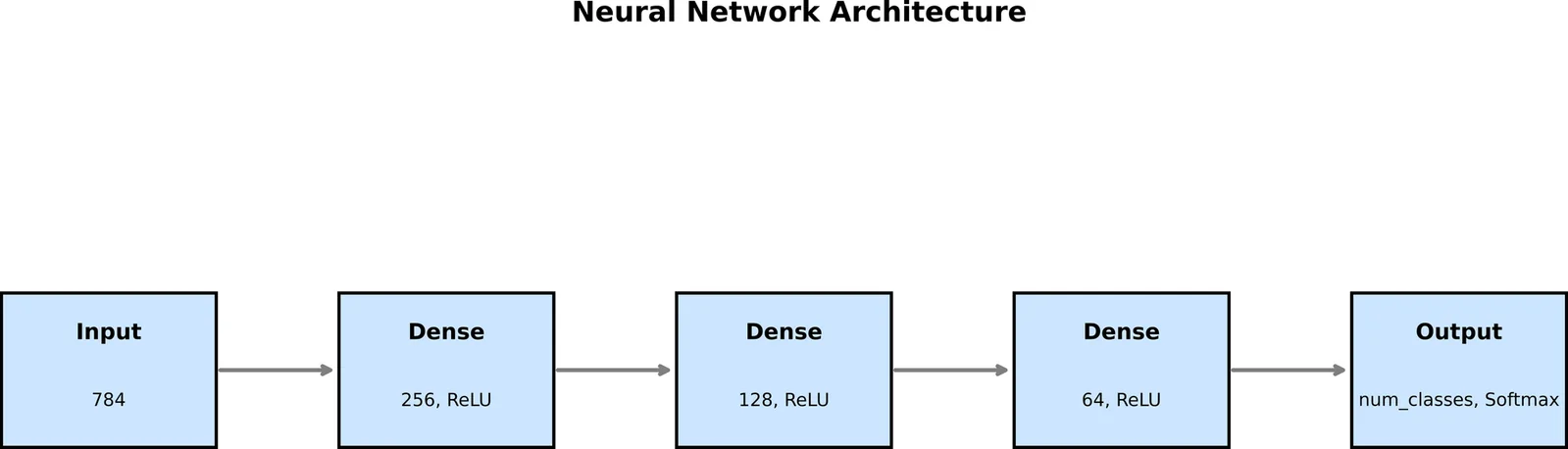
The proposed Self-Healing neural network (SHNN) neural network architecture, where the neural network has three dense layers and the input layer and the output layer.

**Fig. 3:**
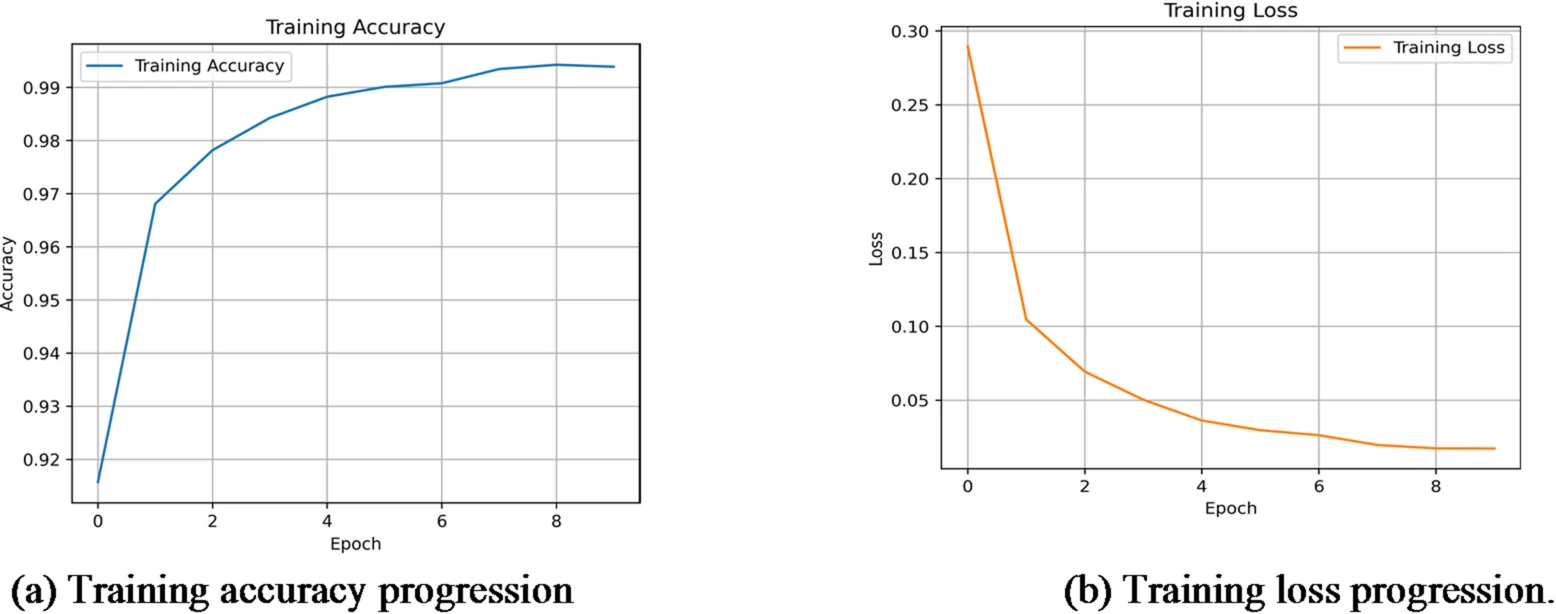
Baseline model training performance over 10 epochs. (a) Accuracy progression, (b) Loss progression.

The SHNN consists of a feedforward neural network that is being learnt on the MNIST dataset of digit recognition. The MNIST dataset consists of 70,000 grayscale handwritten digit images of size 28 × 28 pixels. For the experiments conducted in this work, the dataset was partitioned using an 80:20 train-test split with random_state = 42 to ensure reproducibility (handwritten digits). which are grayscales of size 28 × 28 pixels. To make them easier to compute with, the flattening of each image into a 784-dimensional input vector is taken. These 784-dimensional vectors are fed on to the network architecture input layer. The three fully connected (Dense) hidden layers, i.e. dense0, dense1 and dense2, are then used which undertakes the ReLU activation function to introduce non-linearity. The last layer is a Dense layer that contains 10 neurons and softmax activation, which gives class position of the 10 categories of digits (0–9) (Figs. 1, 2 and 3).

Categorical cross-entropy loss combined with Adam is used to train the model. A short period of training (e.g. 10 epochs) is carried out to determine a baseline of classification accuracy. The weights learned and mid-layer outputs are stored to be used as the reference to the healing process (Tables 1, 2).

**Table 1:** Summary of the base neural network applied in the SHNN model.

Layer	Type	Units	Params
input_layer	Input	784	0
dense0	Dense	256	200,960
dense1	Dense	128	32,896
dense2	Dense	64	8,256
output	Dense (Softmax)	10	330

It has three fully connected hidden layers of dimensionalities that gradually diminish in size (256 − 128 -64) and then there is the softmax classification layer. This small-scale design enables the simulation of structural damage and effective patch repair to be controlled. Total parameters ≈ **242**,**762**.

### Formal definition of damage models

In order to rigorously capture the failure conditions that SHNN framework deals with, we model structural corruption as well as adversarial perturbation. These definitions give a single mathematical abstraction of internal degradation of parameters and externally induced manipulation of inputs that are in line with previous results in adversarial robustness^23,24^ and neural fault analysis^25^.

**Table 2:** Notion table for symbols.

Symbol	Meaning
x	Input sample
y	Ground truth label
W	Original network weights
W′	Corrupted weights
f(x)	Original network mapping
f′(x)	Damaged network
\epsilon	Adversarial perturbation magnitude

#### Structural corruption model

The Structural Corruption model represents a model created to explain corruption across different scenarios. Parameters Let the trained neural network be parameterized by weights $W \in {\mathbb{R}}^{n}$. The model of structural damage is represented by the action of corruption operator $C\left( \cdot \right)$ on the weight tensor:

$$W^{\prime} = C\left( W \right)$$

where $W^{\prime}$ is the corrupted parameter space.

We take three corruption modes as representatives:

1. **Zeroing (Hard Nullification)**:

$$C_{{{\mathrm{zero}}}} \left( W \right) = 0$$

This simulates disastrous parameter flakeout like memory overwriting, excessive pruning or corruption at the hardware level.

(2) **Additive Gaussian Noise**:

$$C_{{{\mathrm{noise}}}} \left( W \right) = W + {\mathcal{N}}\left( {0,\sigma ^{2} } \right)$$

where *σ*controls corruption magnitude. This approximates soft degradation effects such as quantization noise, thermal instability, or analog drift observed in deployed systems.

(3) **Random Sign Flip (Bit-Level Corruption)**:

$$C_{{{\mathrm{flip}}}} \left( W \right) = W \odot M$$

where $M \in \{ - 1,1\} ^{n}$ is a random binary mask. This abstraction aligns with bit-flip fault injection models shown to critically disrupt neural inference in hardware-level attacks^25^.

Such corruption operators provide a generalized formulation of internal structural degradation in neural networks deployed in long-running or resource-constrained environments^26^.

#### Adversarial perturbation model

Unlike structural corruption, adversarial attacks manipulate the input space while keeping model parameters unchanged. Let *x* denote an input sample with label *y*, and let $J\left( {\theta ,x,y} \right)$ denote the loss function of the network parameterized by *θ .*

**Fast Gradient Sign Method (FGSM)**.

Originally introduced by Goodfellow et al.^23^, FGSM generates adversarial samples via a single-step perturbation in the direction of the gradient of the loss with respect to the input:

$$x_{{{\mathrm{adv}}}} = x + \in \cdot {\mathrm{sign}}\left( {\nabla _{x} J\left( {\theta ,x,y} \right)} \right)$$

where *∈*controls the perturbation magnitude.

**Projected Gradient Descent (PGD)**.

Madry et al.^24^ extended this formulation to an iterative adversary:

$$x_{{t + 1}} = { {\Pi }}_{ \in } \left( {x_{t} + \alpha \cdot {\mathrm{sign}}\left( {\nabla _{x} J\left( {\theta ,x_{t} ,y} \right)} \right)} \right)$$

where:

- *α* is the step size,
- ${{\Pi }}_{ \in }$ projects the perturbed sample into an *∈*-bounded norm ball.

PGD is widely regarded as a strong first-order adversary and serves as a robustness benchmark in adversarial defense research^24^.

#### Unified damage perspective

Under this formulation:

- Structural damage modifies parameters:

$$W \to W^{\prime}$$

- Adversarial damage modifies inputs:

$$x \to x_{{{\mathrm{adv}}}}$$

Both transformations alter internal activations, which SHNN detects via layer-wise activation discrepancy analysis. Unlike continual learning approaches that update global parameters to maintain performance^27^, SHNN performs localized compensation through modular patch functions, preserving unaffected representations. This localized strategy enables cross-domain adaptability, aligning conceptually with broader domain generalization principles^28^.

### Structural damage simulation

To test the trained model with internal problems, artificial damage is caused. These model damage are akin to actual issues, e.g., hardware failures, memory corruption, unjustified network pruning, or quantization errors. These defects are added using the apply_structural_damage() function, which randomly selects one of the four levels (dense0, dense1, dense2 or the output layer) and applies one of two damage mechanisms. When the layer biases and weights are rendered to zero in the zero-out damage mode, the layer produces a constant output therefore cancelling out whatever has been learnt in the zero-out damage mode. The weights of the layer are perturbed under the random mode, whereby the perturbation is done on the weights randomly by using small values in a uniform distribution (U(− 0.05, 0.05)), which makes it appear as if noise is being added or the system is being reset by accident. This simulation of structural damage is a stand-in for problems that could happen during real-world deployment that are not predicted. Structural damage disturbs internal calculations, making localized diagnostics and patch-based repair applicable recovery procedures. Adversarial inputs, on the other hand, influence the model from the outside.

### Damage localization through activation comparison

*Damage localization using activation differences*

For finding out the faulty part, SHNN measures the differences between activations of the clean (baseline) and faulty neural network layers. For each layer * l*, the deviation score is computed using Mean Absolute Error (MAE):

$$\:{D}_{l}=\mathbb{E}[\mid\:{A}_{l}^{clean}-{A}_{l}^{damaged}\mid\:]$$

where $A_{l}$denotes the activation of layer *l .*

The faulty layer is chosen as:

$$l^{*} = {\mathrm{arg}}\mathop {{\mathrm{max}}}\limits_{l} D_{l}$$

The damaged layer is selected strictly as the layer with the maximum MAE deviation (argmax), ensuring a consistent and reproducible localization rule across all experiments.

### Healing structural defects via modular patches

After the detection of the defective layer, the modular patch is added to the model to recover the output of the compromised layer without affecting the other layers of the model. The modular structure of the patch is based on the principles of biological self-healing, as well as the use of malleable structures for machine learning. It is usually implemented as a lightweight neural network, usually with one or two dense layers, which is placed immediately before the defective layer. The main goal of the modular patch is to transform the input received by the defective layer in order for the subsequent computation to mimic the original, healthy model.

During the training of the modular patch, the input received by the defective layer from the healthy, pre-damage model is used as the input for the patch, whereas the pre-damage activations are used as the target output. To ensure that the patch is the only component that learns, all the other layers of the model are frozen, which prevents the weights of the other layers from being updated. Therefore, the goal of the modular patch is to minimize the difference between its output and the original activation. As the loss function, The patch is trained to minimize reconstruction error using Mean Squared Error (MSE), while MAE is used exclusively for damage localization. Once the training of the modular patch is complete, the defective model is evaluated, healing the original model without the need for resetting the entire model. Thus, MAE is used for localization, while MSE is used solely as an optimization objective during patch training.

### Patch architecture design criteria

The architecture of the repair patch is an important criterion that must be satisfied to ensure an optimal trade-off between accuracy and computation cost. In the context of the proposed SHNN, the repair patch is implemented as an efficient feedforward network that approximates the activation mapping of the compromised layer.

A number of criteria have been established to guide the choice of the architecture for the repair patch:

**Layer compatibility**: The input and output dimensions of the repair patch must be compatible with the interface of the compromised layer. As such, the repair patch accepts the same input tensor that was processed by the compromised layer. Moreover, the output of the repair patch also has the same dimensionality as the activation of the compromised layer.

**Minimal parameterization**: To ensure the lightweight nature of the repair mechanism, the architecture of the repair patch must have minimal parameterization. In the context of the proposed method, the architecture of the repair patch consists of one or two Dense layers with ReLU activation functions.

**Localized optimization**: To ensure the localized optimization of the repair patch, the original network weights remain frozen during the optimization process. Only the weights of the repair patch are updated during the optimization process.

**Sensitivity analysis**: To establish the optimal number of training samples for the optimization of the repair patch, an ablation study was conducted. As shown in Table 3, the accuracy of the proposed method increases rapidly for an increasing number of training samples for the repair patch. However, the accuracy increases marginally for values between 5,000 and 10,000.

**Table 3:** Patch training sample ablation study.

Patch samples	Healed accuracy (%)	Patch training time (s)
1,000	93.76	3.75
5,000	97.13	4.82
10,000	97.51	3.95
20,000	97.73	6.52

This is important, as it shows how we have to find the right balance between how fast we can recover from damage and how accurate we can recover. By working with smaller sets, we can get patch convergence to happen much faster, but our accuracy will fall by a small amount. By working with larger sets, we can increase our accuracy by a small amount, but we will increase our patch recovery time.

*Design Trade-off*

In theory, we could increase our patch architecture size to be larger, allowing us to recover our damaged representations with greater accuracy. However, by doing so, we will increase our inference costs and risk overfitting our patch architecture. Therefore, we stick with our small patch architecture, allowing us to recover quickly and with low system costs.

These design choices allow the patch module to approximate the damaged representation while preserving the efficiency advantages of localized neural repair.

### Theoretical perspective on patch-based representation recovery

We can view deep neural networks as stacks of functions, each composed of a series of non-linear transformations, where each subsequent layer transforms our input features into higher-level representations. If we compose our neural network as a series of functions:

$$f\left( x \right) = f_{L} \left( {f_{{L - 1}} \left( { \ldots f_{2} \left( {f_{1} \left( x \right)} \right)} \right)} \right)$$

with f_i_(·) being the transformation applied to the i-th layer. Each layer contributes to the construction of intermediate features that eventually contribute to the prediction accuracy.

When the network is impaired either structurally or subjected to adversarial perturbations, the construction of the hierarchical representation is impaired. Specifically, when the k-th layer is impaired, the transformation f_k_(·) is impaired and produces incorrect activations. The impaired network is equivalent to the following:

$$f^{\prime}\left( x \right) = f_{L} \left( { \ldots f_{{k + 1}} \left( {f_{k}^{{{\prime}}} \left( {f_{{k - 1}} \left( { \ldots f_{1} \left( x \right)} \right)} \right)} \right)} \right)$$

where $f_{k}^{\prime }$ is the impaired transformation of the k-th layer. The impaired transformations will affect the overall prediction accuracy of the network since the latter layers depend on the intermediate features provided by the former ones.

The proposed SHNN method compensates for the impaired localized transformation through localized functional compensation. Instead of retraining the impaired network, the proposed method employs a patch layer $P_{\theta } \left( \cdot \right)$, located before the impaired layer. The patch layer is designed to compensate for the impaired transformation of the k-th layer by learning the original transformation of the impaired layer:

$$P_{\theta } \left( {z_{{k - 1}} } \right) \approx f_{k} \left( {z_{{k - 1}} } \right)$$

where z_k_−1 is the activation output from the preceding layer.

The concept is supported by a strong theoretical foundation, as the universal approximation theorem asserts that feedforward networks of sufficient capacity are able to approximate any continuous function on a bounded domain. This implies that a compact neural module is, in principle, able to replicate the necessary mapping to recover the original form of the damaged layer.

The concept of deep networks as representation learners implies the development of abstraction layers, with each layer heavily relying on the structured outputs of the preceding ones. Therefore, if a layer is damaged and affects the abstraction representations, it is possible to recover the functionality of the network by repairing the correct mapping of adjacent layers, rather than having to relearn the entire network.

Additionally, research on the robustness and pruning of neural networks suggests that networks are, to some extent, redundant and resilient to parameter perturbations.

Further, the concept of patch-based repair has also been investigated in the context of the recently proposed repair frameworks, where additional modules are incorporated to correct the faulty behaviors while the parameters are kept the same. However, the SHNN method extends this concept further by identifying the faulty regions using activation discrepancy analysis and subsequently learning the corrective mappings to account for the faulty internal transformations.

Further, the role of domain statistics in this context is also important, as the cross-domain representational transfer is influenced by the statistics of the features in the domain. In fact, studies on domain generalization have also shown that the learned representations are a mixture of the invariant and the dataset-specific features. In this context, the corrective mappings learned by the patch-based modules are likely to work well within the domain, but the generalization across different domains is questionable.

Moreover, the success of the localized repair is also influenced by the architectural properties, including the network depth, the presence of nonlinearities, and the level of abstraction in the features. In fact, the initial layers are likely to represent low-level features, whereas the later layers represent higher-level features. In this context, the accuracy loss due to damage in the later layers is higher, but the redundancy in the features learned in the later layers is also higher, which makes the compact patch-based mappings an effective approximation of the faulty mappings.

Overall, the SHNN repair strategy can be interpreted as learning a localized corrective transformation that compensates for the corrupted layer function. By freezing the original network parameters and optimizing only the patch module, SHNN performs targeted representation recovery while maintaining minimal computational overhead.

### Simulation of adversarial attack and recovery

SHNN also addresses adversarial attacks, which are external manipulations designed to deceive the neural network through subtle, often imperceptible perturbations. Two attack tactics are used to test the model’s robustness. The first one is the Fast Gradient Sign Method (FGSM). This is a single-step gradient-based assault that changes the input pixels in the direction that makes the model loss the most. The second is the Projected Gradient Descent (PGD) assault, which is an iterative form of FGSM that makes minor changes over and over again while keeping them within a defined ϵ-ball around the initial input. This makes it stronger and harder to fight against. Adversarial inputs mostly change the internal activations of a model instead of its weights. The SHNN framework compares activations between clean and corrupted inputs to detect these problems. The layer with the most significant average activation variation is the most affected and is chosen as the target for patch-based mending. This method lets SHNN quickly find and fix damage caused by adversaries without retraining the entire network.

### Healing adversarial damage

The method for healing antagonistic damage is very similar to fixing structural problems, but there are a few other factors to consider. During patch training, a dataset that includes both clean and adversarial samples is used to ensure balanced learning. A lightweight patch network is inserted before the most affected layer identified through activation deviation analysis. The patch is trained to restore clean-like activations when the model is exposed to adversarially perturbed inputs, using the same loss function as in structural repair using Mean Squared Error (MSE) loss for activation reconstruction but explicitly applied to adversarial activations. After training, the patched model is tested on both clean and hostile test sets to assess its increased robustness. This method helps the network create more robust internal representations, allowing it to handle adversarial noise while remaining very accurate on data unaffected by it.

### System integration and graphical interface

All components of the SHNN methodology are integrated together using an interactive GUI implemented using Streamlit. The GUI is user-friendly and allows for easier experimentation and analysis using the SHNN methodology. Using the GUI, you can upload your dataset in the form of a .mat file, train the base neural network, perform adversarial attacks, and train/insert the healing patches. Additionally, you can visualize the performance metrics and layer activations in real time using the GUI, giving you a good idea of how the healing is proceeding. Using the GUI, you can visualize and compare the accuracy, patch training loss, and pre- and post-healing performance side by side, with the visuals generated using Matplotlib and Seaborn libraries.

### Recovery metric definition

To evaluate the efficacy of self-healing, we define Recovery (%) metric as the percentage of accuracy recovered using after self-healing compared to the percentage of accuracy reduced due to adversarial attacks. Let $\:{A}_{b}$ be the baseline test accuracy of the undamaged model, $\:{A}_{d}$ be the accuracy after damage or attack, and $\:{A}_{h}$ the accuracy after healing. Recovery is computed as:

$${\text{Recovery~(\% )}} = \frac{{A_{h} - A_{d} }}{{A_{b} - A_{d} }} \times 100$$

The Recovery metric gives us an idea of how much of the reduced accuracy is recovered using self-healing. An ideal Recovery of 100% indicates full recovery of the network, whereas lower Recovery values indicate partial recovery of the network.

### Data splitting strategy and experimental reproducibility

For the sake of reproducibility and consistency among all experiments, a fixed and deterministic data splitting procedure was used for all datasets considered in this paper.

**MNIST**:

The MNIST dataset comprises 70,000 grayscale handwritten digits images of dimensions 28 × 28 pixels, which contain 60,000 training and 10,000 testing images. All images were rescaled to the range [0,1] through division by 255.

An 80:20 train-test split was used for the datasets for performing structural damage and adversarial recovery experiments via train_test_split function in Scikit-learn with random_state = 42 to ensure deterministic reproducibility.

In addition to the aforementioned split, sub-samples from the training set were used for patch-training experiments. For base model training, 15,000 training samples were used, whereas for patch-healing experiments, the number of samples varied from 1,000 to 20,000 samples based on the ablation study design.

For adversarial retraining experiments, an additional validation set of 10% was created during patch optimization via Keras validation_split = 0.1.

**Fashion-MNIST**:

The Fashion-MNIST dataset was used to evaluate cross-domain transfer performance using the original benchmark split of 60,000 training samples and 10,000 testing samples. All images were reshaped to 784-dimensional vectors and normalized to the range [0,1].

**Sign Language MNIST**:

The Sign Language MNIST dataset was used to evaluate the adaptability of the SHNN framework on gesture-based handwritten sign recognition tasks. The dataset was normalized to the range [0,1] and partitioned using an 80:20 train-test split with random_state = 42 to ensure reproducibility and deterministic evaluation across experiments.

**CIFAR-10**:

For the CNN experiment, the CIFAR-10 dataset was used with the usual benchmark split of 50,000 images for training and 10,000 images for testing. The RGB images were normalized to [0,1].

**Reproducibility of experiment**:

Random seeds of NumPy, TensorFlow, and Python were kept constant in the statistical experiment setup. The structural damage simulation was done in the experiment setup with the random seeds. Argmax based MAE criterion was used for layer localization.

## Evaluation, analysis, and findings

### Overview of experimental setup

We ran all tests in Python using TensorFlow 2 and the Self-Healing Neural Network (SHNN) framework. The basic model was a regular feedforward, fully connected deep neural network that was trained on the MNIST dataset. It consisted of an input layer with 784 units (flattened 28 × 28 images), three hidden Dense layers with 256, 128, and 64 units respectively, and a final softmax output layer with ten classes. All hidden layers employed the ReLU activation function, while the output layer used softmax to compute class probabilities. The model was trained for 10 epochs using categorical cross-entropy loss and the Adam optimizer with a learning rate of 0.001 and a batch size of 128. Two types of performance degradation were evaluated: **adversarial attacks** and **structural damage**. The Fast Gradient Sign Method (FGSM) with ε = 0.2 was used to make single-step perturbations for adversarial testing. The PSO-guided PGD attack employed epsilon 0.2, step size 0.01, and 30 iterations to generate stronger iterative attacks. To simulate structural damage, they either set weights of a chosen Dense layer to 0 or introduced random Gaussian noise with sigma 0.1. In each case, SHNN’s damage detection module compared activation between undamaged and damaged models through Mean Absolute Error(MAE) to identify which layer is most impacted by damage. This impacted layer is then replaced with a new patch trained on 10,000 samples with the same optimizer and loss function to facilitate fast learning. In terms of performance, accuracy is measured over three stages: pre-damage accuracy, post-damage accuracy, and post-healing accuracy. Each experiment is run 10 times, with randomized damage location and perturbations to guarantee statistical accuracy.

The number of epochs for each experiment is varied between structural healing and adversarial healing. In the case of structural damage, parameters are corrupted deterministically, allowing for fast learning with fewer epochs. In contrast, adversarial attacks must be made robust against constantly changing perturbations, which requires longer epochs to converge. Each number is chosen based on empirical results to guarantee convergence without overfitting.

Statistical reliability was evaluated by repeating each experiment across multiple random seeds. The reported results correspond to mean values with standard deviations, ensuring that the observed improvements are consistent across runs.

**Adversarial evaluation protocol**

Tests for structural damage are carried out using the whole test set (10,000 instances).

The adversarial testing is done on a constant subset of the first 100 test samples (X_test[:100]). These samples are always used for adversarial generation and layer-wise activation mismatch computation for damage localization.

Accuracy in clean tests for adversarial tables refers to these 100-samples only, while the rest of the tests have been performed using the whole test dataset.

Tests involving several random seeds are represented as mean ± standard deviation, whereas a single-valued table represents tests done on one seed.

### Baseline model performance

The SHNN baseline was trained on MNIST for 10 epochs with batch size 128, categorical cross-entropy loss, and the Adam optimizer. A short training period was chosen to allow rapid simulation of multiple damage and healing cycles efficiently.

The baseline model after training yielded the following performance (Table 4).

**Table 4:** Baseline model performance.

Metric	Value (%)
Training accuracy	99.43
Test accuracy	97.76

Accuracy computed on the full test set (10,000 samples).

**Table 5:** Structural damage recovery performance of SHNN.

Method	Baseline accuracy (%)	Damaged accuracy (%)	Healed accuracy (%)	Recovery (%)	Repair time (s)	Memory overhead (MB)
SHNN (Proposed)	97.76	9.19	95.76	97.74	3.07	0.93

Memory overhead refers strictly to patch module parameters.

Table 5 reports the recovery performance of the SHNN framework under structural corruption. The damaged model exhibits a severe degradation in classification accuracy, dropping from 97.76% to 9.19%. After applying the localized healing patch, accuracy is restored to 95.76%, approaching the original baseline performance of 97.76% due to patch fine-tuning effects. The repair process requires approximately 3.07 s and introduces minimal memory overhead.

Despite the brief training timeframe, the model achieved 99.43% accuracy on the training set and 97.76% on the test set, providing a solid benchmark for comparison against structural and adversarial damage as well as post-healing performance.

**Table 6:** Patch training sample ablation study.

Patch samples	Healed accuracy (%)	Patch training time (s)
1,000	93.76	3.75
5,000	97.13	4.82
10,000	97.51	3.95
20,000	97.73	6.52

**Fig. 4:**
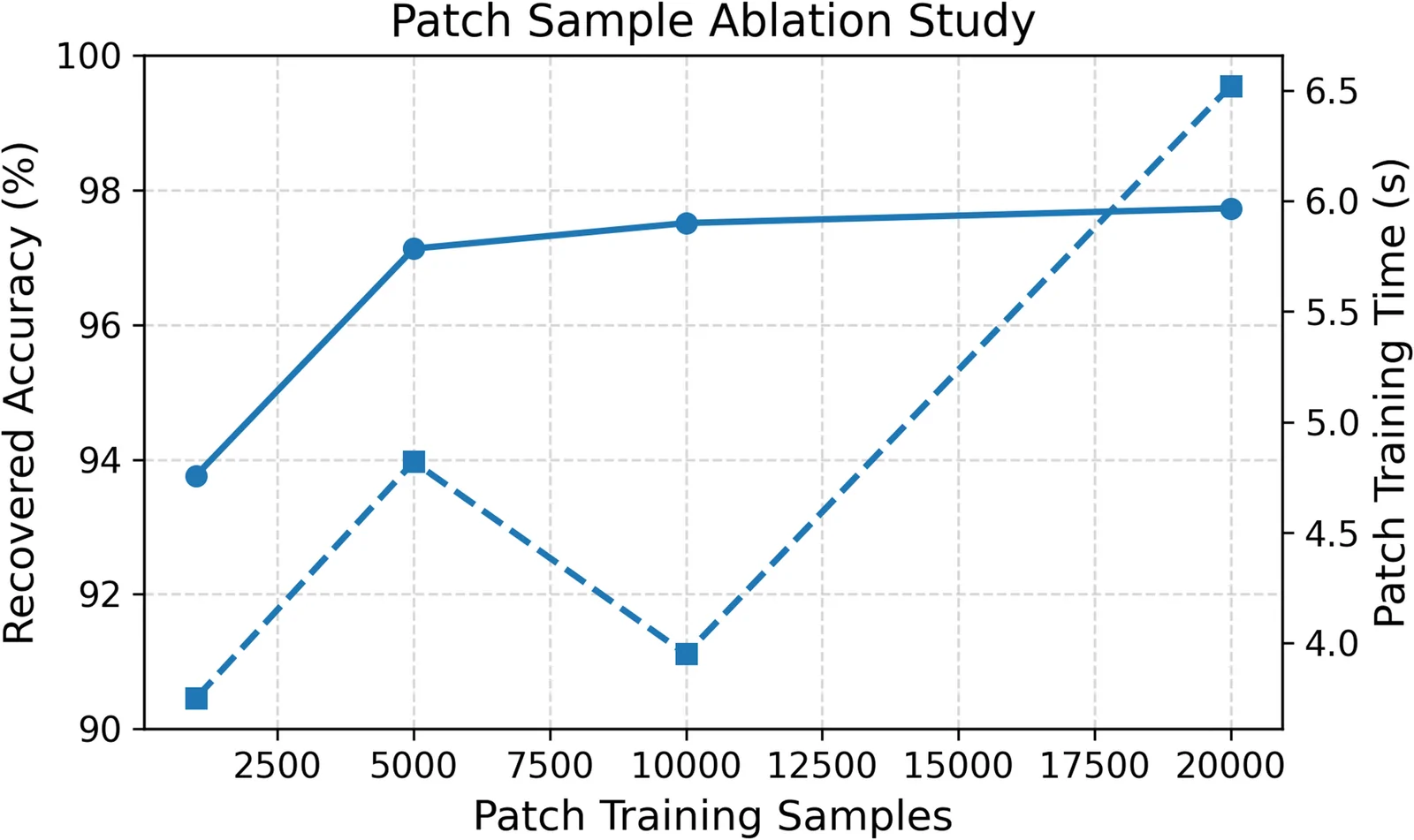
Patch sample ablation showing recovered accuracy and patch training time.

In order to examine patch-based repair data efficiency, an ablation experiment was performed by changing the size of the samples utilized to train the healing patch. The accuracy of recovery increases at a fast rate with increase in samples and levels off at about 5k-10k samples as indicated in Table 6; Fig. 4. Out of this range, the marginal gains are exhibited and the repair time increases. This shows that SHNN can attain close to full recovery with a small set of data hence adaptable in quick and low overhead deployment conditions.

### Impact of adversarial attacks

Two classical adversarial attack strategies—Fast Gradient Sign Method (FGSM) and Projected Gradient Descent (PGD)—were applied on the trained baseline model to evaluate SHNN’s robustness. Each attack was performed on a fixed subset of the first 100 test samples (X_test[:100]).

The same fixed subset is used for both clean and adversarial evaluations to ensure fair and consistent comparison.

Preliminary experiments across different perturbation strengths (ε ∈ {0.1, 0.15, 0.2}) showed consistent layer localization and similar recovery trends, indicating that SHNN’s repair mechanism is stable across moderate variations in attack strength.

FGSM Attack Results: Test accuracy dropped significantly under FGSM perturbations (ε = 0.2), as summarized in Table 7.

**Fig. 5:**
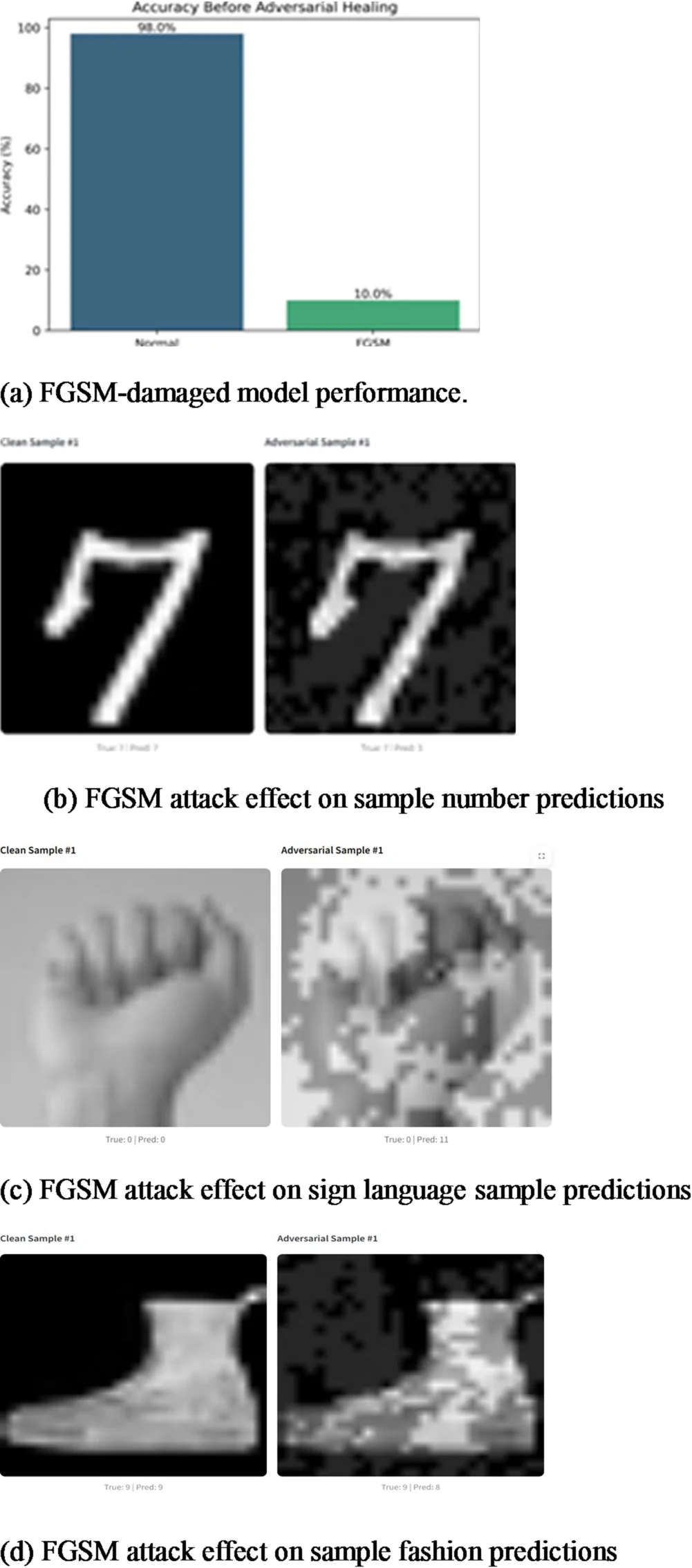
Accuracy comparison on test samples under FGSM attack (ε = 0.2). (a) Model under attack, (b) FGSM Attack on Numbers(Clean vs. Adversarial), (c) FGSM Attack on Sign Language (Clean vs. Adversarial), (d) FGSM Attack on Fashion Symbols(Clean vs. Adversarial).

**Table 7:** Accuracy (%) on 100-sample adversarial subset under FGSM attack.

Evaluation condition	Accuracy (%)
Clean (100-sample subset)	98
FGSM-Adversarial	10

There was an 88% drop in accuracy under the FGSM attack, suggesting vulnerability to small, localized noise-crafted perturbations. Healing mechanisms, however, significantly restored accuracy as seen in Fig. 5.

PGD Attack Results: PGD (ε = 0.20, α = 0.01, 30 iterations) is more invasive and causes even worse performance degradation in the model, as shown in Table 8.

**Table 8:** Accuracy (%) on 100-sample adversarial subset under PGD attack.

Evaluation condition	Accuracy (%)
Clean (100-sample subset)	100
PGD-Adversarial	0

**Fig. 6:**
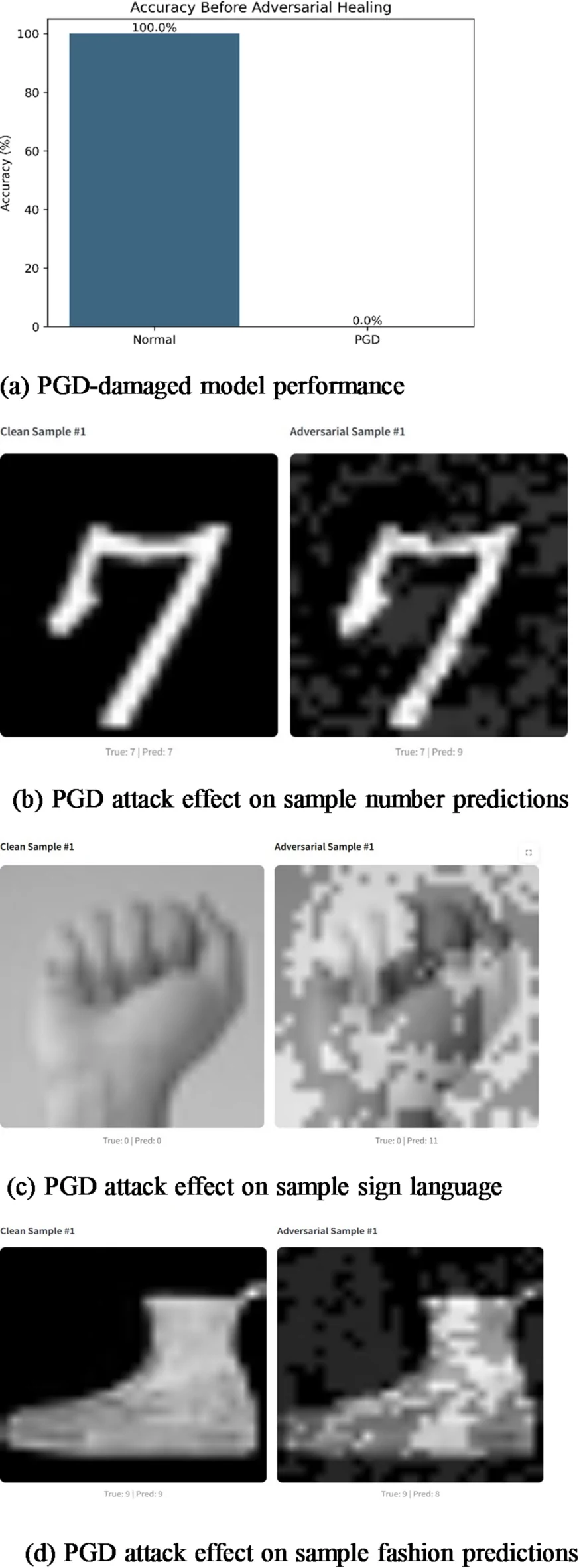
Impact of PGD attack (ε = 0.20, α = 0.01, 30 iterations): (a) Accuracy degradation on test samples, (b) PGD Attack on Numbers (Clean vs. Adversarial), (c) PGD Attack on Sign Language (Clean vs. Adversarial), (d) PGD Attack on Fashion Symbols (Clean vs. Adversarial).

The iterative PGD attack, designed to increase perturbation within limits, reduced classification accuracy to 0%. Figure 6(b), (c), and (d) indicate that adversarial perturbations not only trick the model but also make photos look much different from FGSM, leading to more serious misclassifications.

Layer Damage Analysis (Adversarial Path): Table 8 shows, the most impacted layers after the attack were determined by computing the layer activation discrepancy measure (Mean Absolute Error, MAE) using the get_damaged_layer() function.

**Table 9:** Layer damage scores (MAE).

Layer name	FGSM damage score	PGD damage score
Input	0.013	0.018
Dense0	0.19	0.29
Dense1	0.98	1.90
Dense2	5.00	12.00
Output	0.17	0.20

**Fig. 7:**
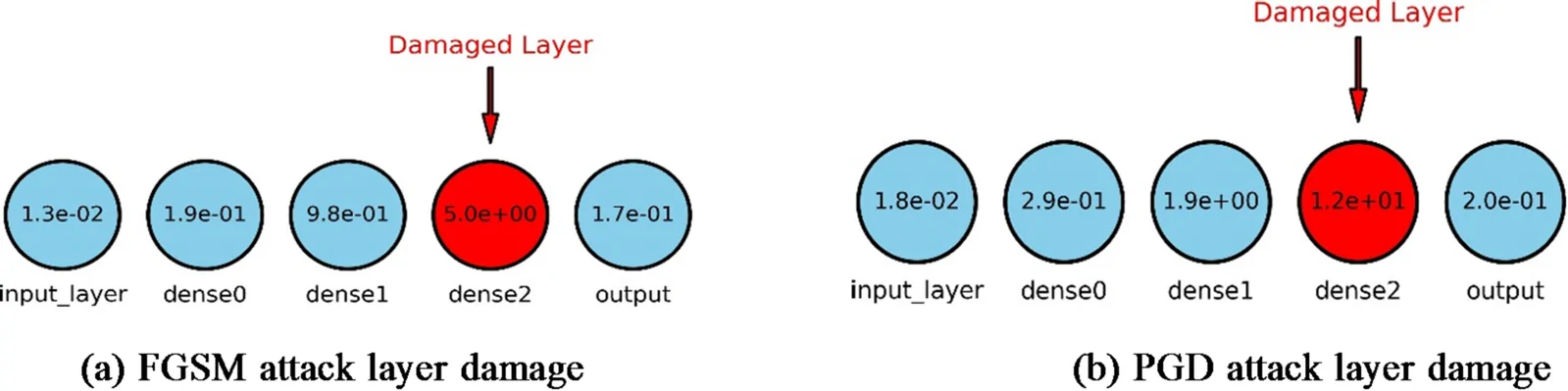
Layer damage visualizations comparing FGSM vs. PGD attacks. Red indicates the most damaged layers based on MAE values.

Dense2 was the most sensitive layer to targeted pixel perturbations in both FGSM and PGD attacks, indicating that mid- to late-feature-extraction stages are the most vulnerable when the model fails.

### Adversarial recovery results

For each adversarial attack scenario, SHNN applied its self-healing mechanism to the layer most severely affected. To keep weight updates to a minimum, all network layers, save the one that was broken, were frozen during this operation. A new, lightweight patch network was created to replace the compromised layer. This new patch network was trained independently for 10 epochs on 50% clean data and 50% adversarial data. By focusing on repairing the compromised area, this approach enabled the model to quickly bounce back and recover performance without requiring retraining of the entire model (Table 9).

**Fig. 8:**
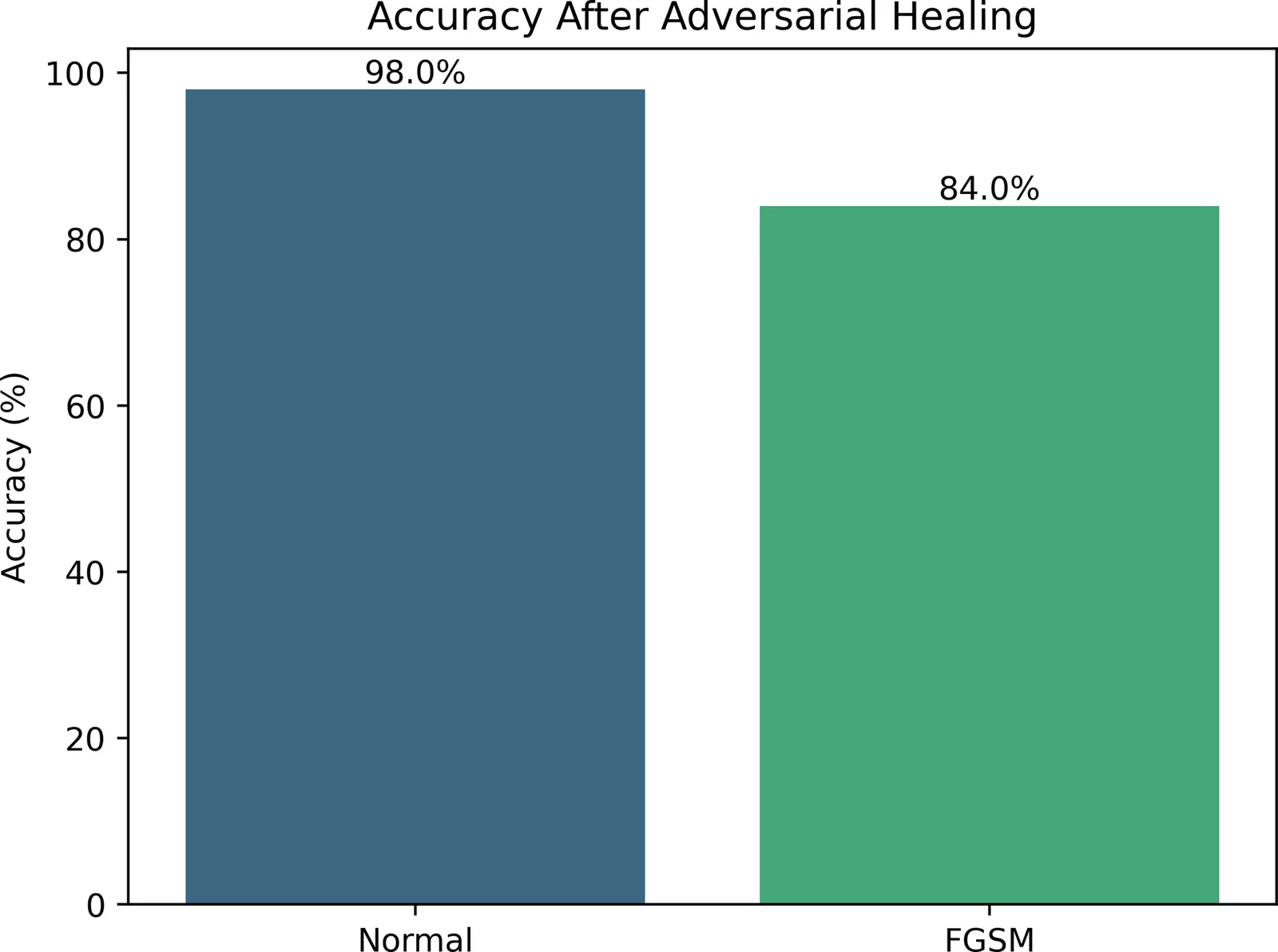
Healed model accuracy comparison: Normal vs. FGSM attack.

**Fig. 9:**
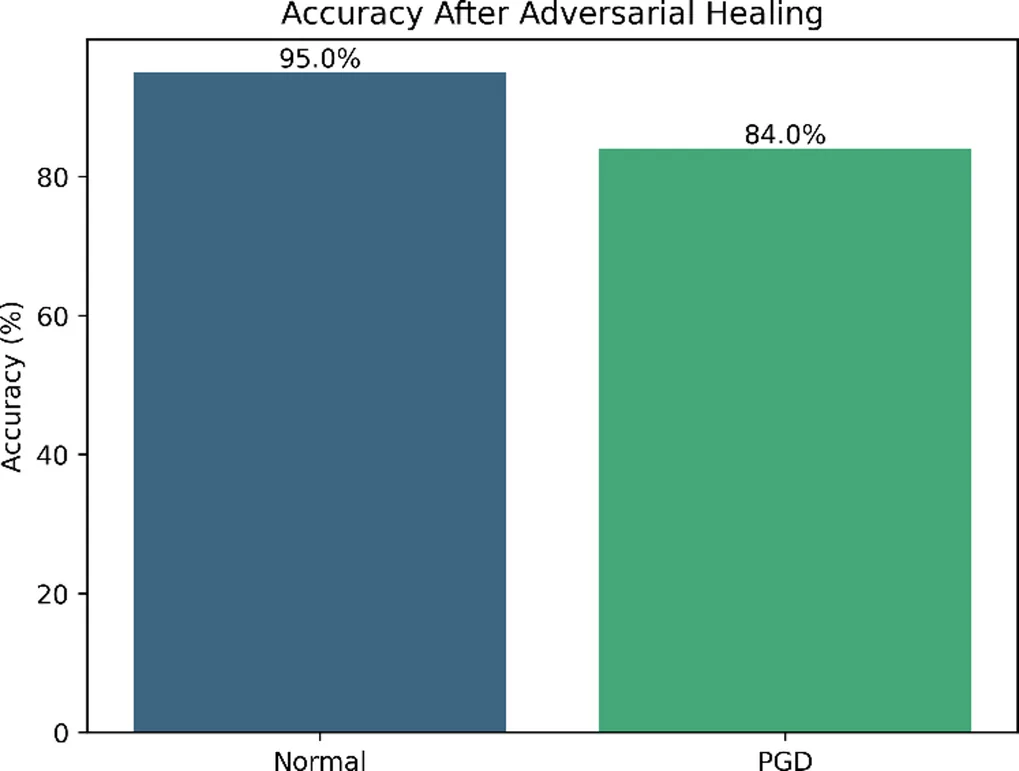
Healed model accuracy comparison: normal vs. PGD attack.

**Fig. 10:**
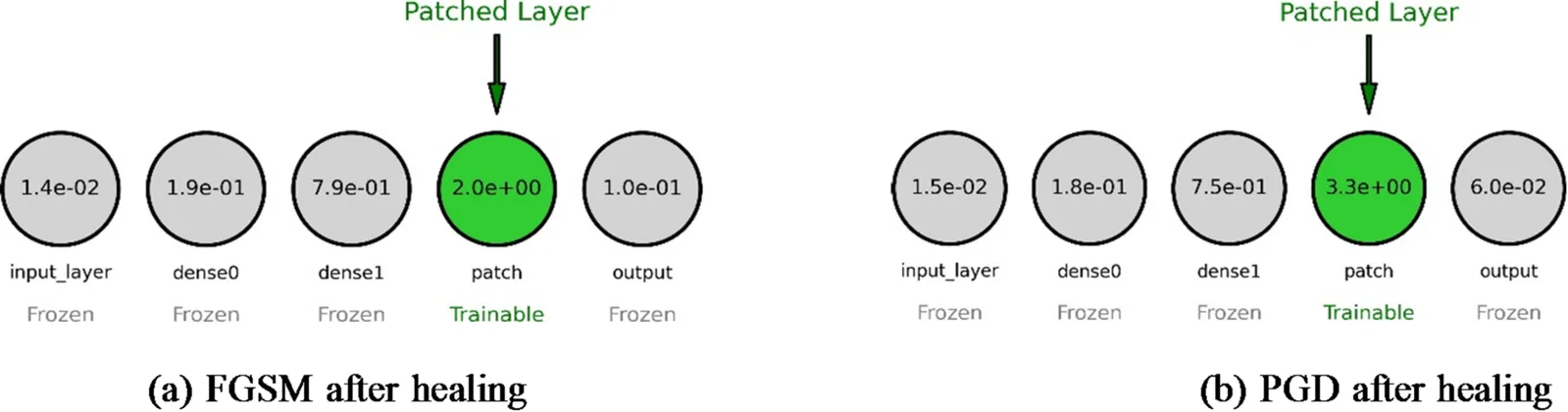
Layer visualization after healing: MAE of layer activation differences between standard and healed model (Green = patch layer).

FGSM Recovery Results: Table 10 presents how much performance is recovered by repairing the dense2 layer, which was compromised the most by the FGSM attack (Figs. 7, 8, 9 and 10).

**Table 10:** FGSM recovery results after patching dense2.

Evaluation condition	Accuracy (%)
Clean (100-sample subset)	98
FGSM-Adversarial (Healed)	84

The patch-based healing technique achieved the recovery of more than 74% of the lost accuracy for the FGSM perturbed inputs, thus proving the capability of the technique to detect and compensate for the adversarial noise in single steps. In the case of the PGD adversarial noise, the recovery results prove that when the patch offsets the considerable damage caused by the adversarial noise in the dense2 layer, the accuracy increases dramatically, as shown in Table 11.

**Table 11:** PGD recovery results after patching dense 2.

Evaluation condition	Accuracy (%)
Clean (100-sample subset)	95
PGD-Adversarial (Healed)	84

Although PGD is a far more potent attack, the patch mechanism still recovered close to 84% of the lost performance on PGD-perturbed inputs.

Layer Damage Reduction: After healing, the MAE difference scores dropped significantly for dense2 under both attack scenarios, as shown in Table 12.

**Table 12:** Layer damage reduction after healing.

Attack type	Damaged layer MAE (Before)	Damaged layer MAE (After)
FGSM	5.00	2.00
PGD	12.00	3.30

The results demonstrate that localized retraining via patch integration is effective in counteracting adversarial degradation, especially for moderate E values. But the PGD recovery was still slightly lower than FGSM because repeated attacks span a more expansive perturbation search space.

**Table 13:** Single-layer vs. multi-layer patch repair.

Repair strategy	Healed accuracy (%)	Patch training time (s)
Single-layer patch	96.77	1.76
Multi-layer patch	89.39	1.69

**Fig. 11:**
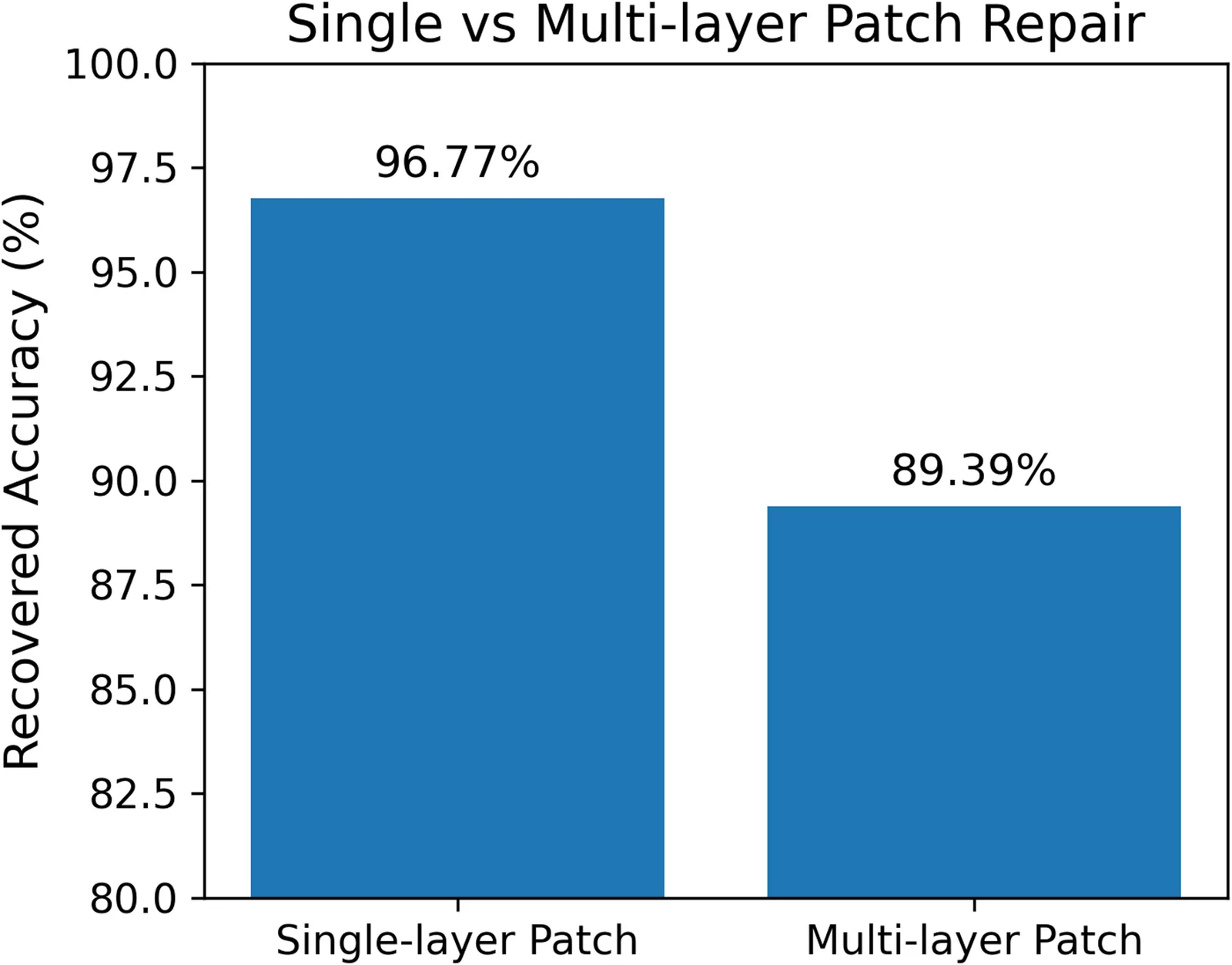
Single-layer versus multi-layer patch recovery performance.

The study was done in comparison of single-layer and multi-layer patching to determine whether a multi-layered repair has any extra advantages. Table 13; Fig. 11 indicate that single-layer patching is always better than multi-layer patching given the same repair time; it is more accurate in the recovery. There is minimal interference with gradient and overfitting, and in multi-layer patching, redundant complexity in optimization is introduced.

### Structural damage simulation

The SHNN architecture was also tested under structural damage scenarios to assess its ability to recover from internal flaws, in addition to adversarial attack evaluations. In these tests, one of two damage mechanisms was used to change the weights of a randomly chosen Dense layer deliberately. When the weights were set to zero, the layer’s contribution to feature extraction was effectively removed. In the randomized mode, the weights were substituted with tiny Gaussian noise (σ = 0.1), which messed up the learnt representations and made it look like something was wrong inside. The apply_structural_damage() method randomly picked one layer and one damage mode each time it ran. This lets the system test its ability to find and fix different types of structural problems.

Zeroing Damage Results: Table 14, in the zeroing case, the functionality learned by this layer was utterly lost. The damaged layer is selected randomly (12, 13, 14, 15, 16, 17 and 18).

**Fig. 12:**
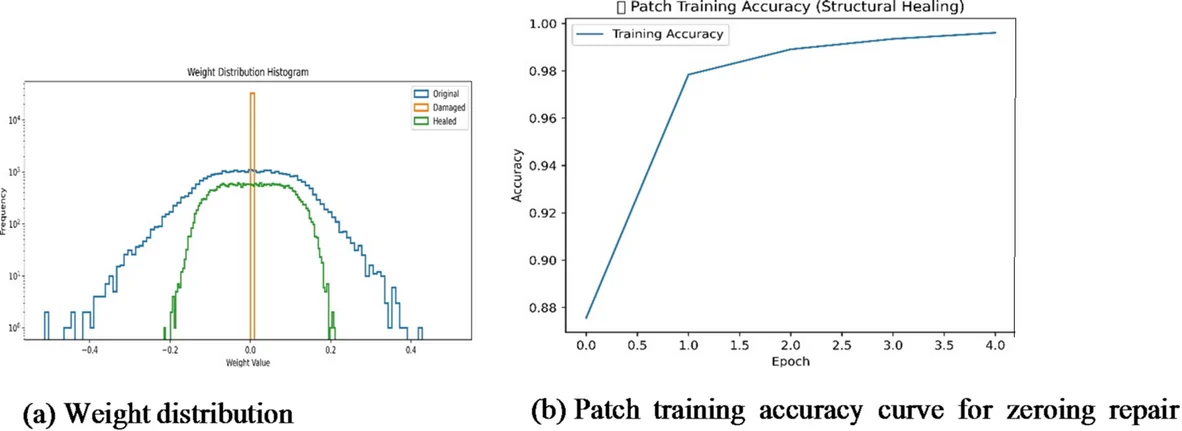
Zeroing damage analysis: weight distribution and healing training accuracy. (a) Weight distribution. (b) Patch training accuracy curve for zeroing repair.

**Table 14:** Zeroing damage results.

Evaluation condition	Accuracy (%)
Clean samples (Pre-Damage)	97.76
Zeroed layer (Damaged)	9.94
Zeroed layer (Healed)	97.33

Zeroing a hidden layer reduced accuracy by ∼88%, but patch replacement restored ∼87% of the lost performance. The patch network in SHNN is lightweight and designed to restore only the damaged layer, allowing it to converge within just a few epochs. Training it longer leads to overfitting on corrupted activations with almost no improvement in accuracy. Hence, four epochs were chosen as the optimal point, providing fast and efficient recovery while aligning with SHNN’s goal of real-time self-healing without full retraining.

Random Weight Damage Results: Gaussian noise on weights destroyed the ability of the target layer to operate on clean semantic features as shown in Table 15.

**Fig. 13:**
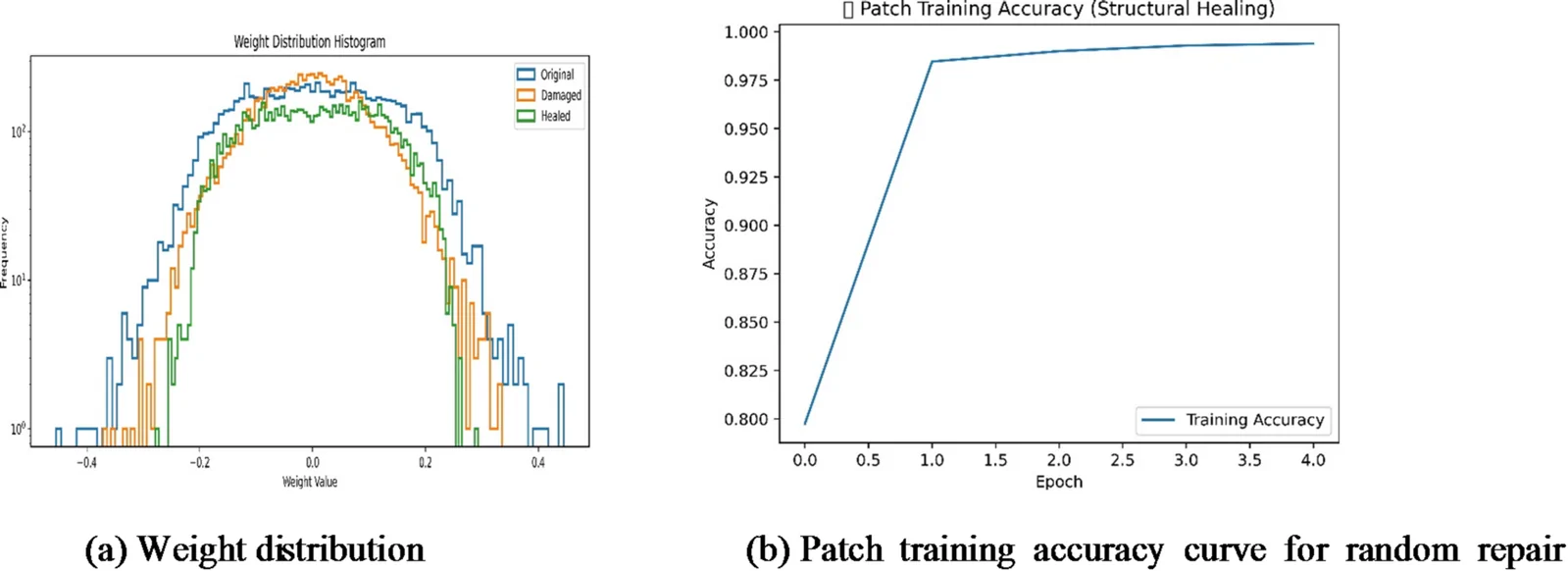
Random weight damage analysis: weight distribution and healing training accuracy.

**Table 15:** Random weight damage results.

Evaluation condition	Accuracy (%)
Clean samples (Pre-Damage)	97.76
Randomized layer (Damaged)	10.33
Randomized layer (Healed)	97.22

Random noise caused slightly less damage than zeroing, but healing still improved performance by ∼87%. The patch network in SHNN is lightweight and designed to restore only the damaged layer, allowing it to converge within just a few epochs. Training it longer leads to overfitting on corrupted activations with almost no improvement in accuracy. Hence, four epochs were chosen as the optimal point, providing fast and efficient recovery while aligning with SHNN’s goal of real-time self-healing without full retraining.

Layer Damage Analysis (Structural Path): The SHNN did not use the conventional Keras intermediate activation extraction method to find structural damage. Instead, the get_layer_outputs() method was used to go through the broken layer by hand for each network layer. The identical clean input batch was fed to both the healthy and broken models, and compare_saved_outputs() computed the Mean Absolute Error (MAE) for each layer.

The damaged layer was defined as the layer with the highest MAE deviation, since corruption in earlier layers can propagate and inflate MAE in later layers.

**Table 16:** Layer damage scores (before healing).

Layer name	Random damage score	Zeroing noise damage score
dense0	0.00	0.00
dense1	0.00	0.83
dense2	2.10	2.10
output	0.17	0.18

Based on the pre-healing results in Table 16, in the case of random damage, dense2 has the highest MAE deviation and is identified as the damaged layer. Under zeroing noise damage, although dense2 had the most significant error (2.10). The damaged layer is identified as the layer with the maximum MAE deviation. In both cases, dense2 is selected as it exhibits the highest deviation.

**Table 17:** Layer damage scores (after healing).

Layer name	Random damage score	Zeroing noise damage score
dense0	0.00	0.00
dense1	0.00	0.78
dense2	1.60	1.00
output	0.00059	0.00086

After the recovery, the MAE deviation values decreased in all the layers. For the layers that had been impaired, the dense2 layer for the random case and the dense1 layer for the zeroing case, the MAE deviation values decreased significantly, while the output layer almost completely recovered, with values between 0.00059 and 0.00086. This shows that the healing process was able to control the propagation of errors.

**Fig. 14:**
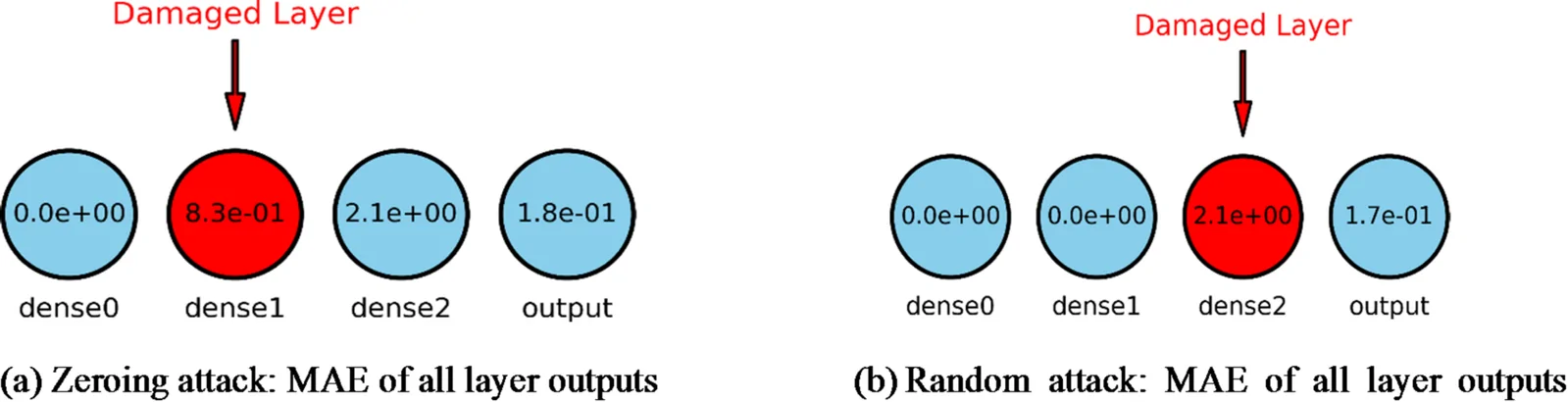
Structural damage visualization of zeroing and random attacks.

**Fig. 15:**
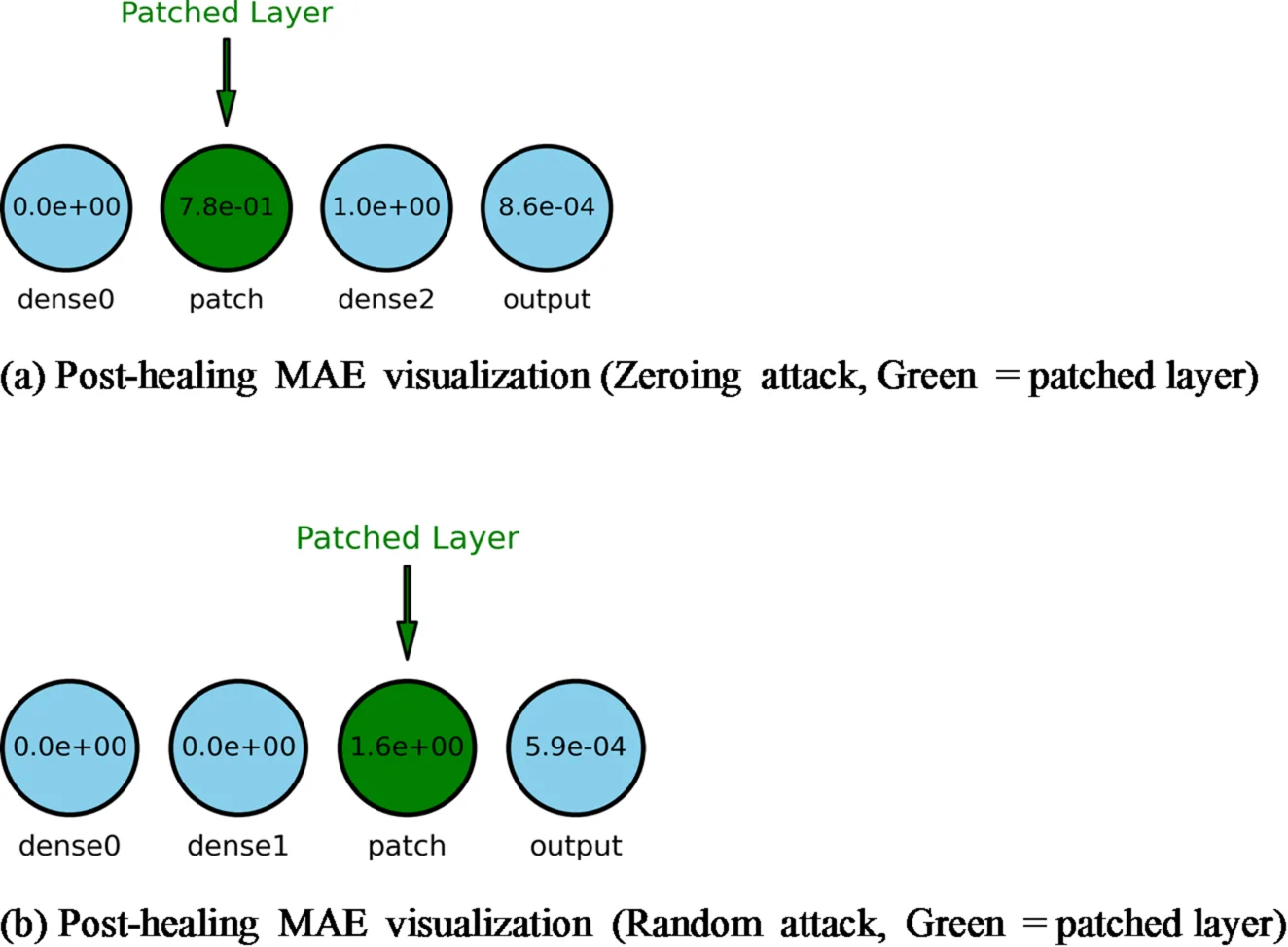
Post-healing structural recovery under zeroing and random attacks.

**Fig. 16:**
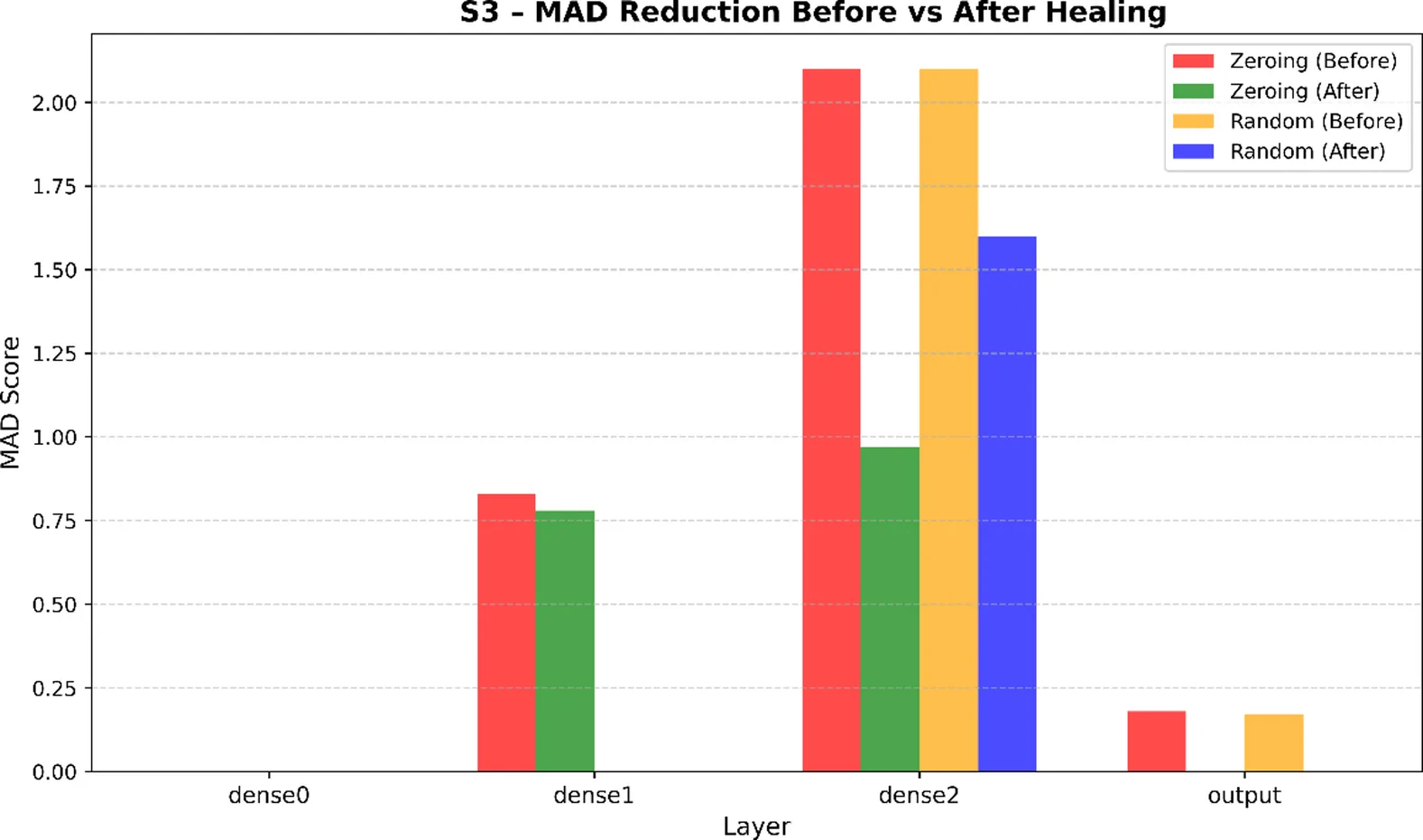
The reduction in MAE before and after the healing process is presented for the zeroing attack and the random attack.

The structural corruption resulting from the zeroing attack results in a significant reduction in accuracy. Nevertheless, the replacement of the targeted patches within the SHNN nearly restores the original performance levels without the need for retraining (Table 17).

## Comparative analysis

The robustness and self-healing potential of the proposed SHNN framework were tested using two types of harm scenarios. The first is the structural harm, whereby the model is subjected to zeroed weights in a single Dense layer or the introduction of random noise within the layer. The second is the adversarial harm, whereby the model is subjected to FGSM and PGD-based adversarial attacks. Table 18 presents the classification accuracy reduction resulting from the various harm types and the efficacy of the healing process.

The structural harm resulted in the highest accuracy reduction, reaching 87–88%. The patch-based healing resulted in the restoration of the model’s performance to within 0.5% of the original accuracy (97.33% vs. 97.76% for the zeroing attack and 97.22% vs. 97.76% for the random noise attack). The adversarial harm resulted in an FGSM-based reduction in accuracy of about 88%. The patch-based healing resulted in the recovery of about 74% of the accuracy loss, suggesting that the model is only partially healed from the adversarial attack. The PGD-based adversarial attack resulted in zero accuracy, but the model was able to recover to about 84% of the original accuracy through the proposed healing mechanism. The structural harm resulted in residual vulnerabilities, whereas the adversarial harm resulted in prolonged harm effects due to the involvement of multiple layers in the model.

**Table 18:** Accuracy drop and recovery across damage types.

Damage type	Pre accuracy (%)	Damaged accuracy (%)	Healed accuracy (%)	Recovery	Healing time	Attack strength / mode
Structural – Zeroing	97.76	9.94	97.33	87.39	3.8	Full weight null
Structural – Random	97.76	10.33	97.22	86.89	3.9	σ = 0.1 Gaussian noise
Adversarial – FGSM	97.76	10	84	74	5.1	ε = 0.2
Adversarial – PGD	97.76	0	84	84	5.4	ε = 0.2, α = 0.01, 30 iters

Healing time represents the amount of time typically required to patch the model back to its original performance level.

Mean Absolute Error (MAE) Score Propagation: In the case of structural damage detection, the MAE is defined as the difference in the output of the pristine model and the damaged model. The damaged layer is identified as the layer with the maximum MAE deviation, thus preventing the propagation of faults to other layers, which could otherwise be mistaken for primary faults. In the case of adversarial attacks, the MAE is defined as the difference in the output of the clean input and the adversarial input. The layers with the highest MAE are marked as damaged. This is because adversarial attacks typically affect multiple layers in the network. In the case of a unified MAE-based maximum deviation rule is used across both structural and adversarial scenarios for consistent damage localization.

Recovery Efficiency: Patch-based healing achieved strong performance restoration across structural damage scenarios. The SHNN framework restored performance to near-baseline levels (97.33% vs. 97.76%) after structural damage. The post-healing accuracies were 97.33% and 97.22%, compared to the baseline of 97.76% for zeroed and randomly disturbed weights, respectively. The system recovered 74–84% of its original performance in adversarial settings, and after both FGSM and PGD attacks, it achieved 84% accuracy. These results show that structural defects can be almost entirely fixed and that the system is quite strong against attacks.

**Fig. 17:**
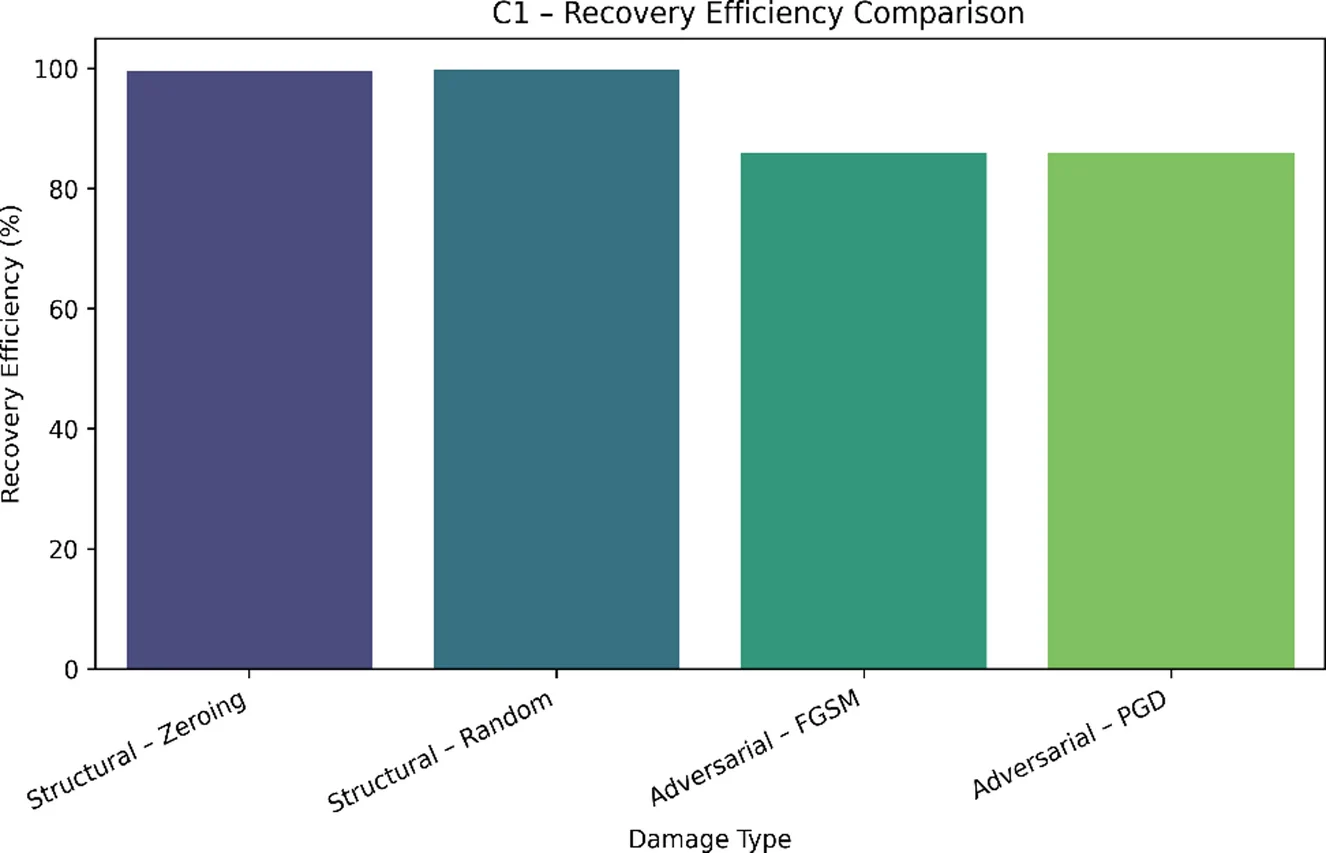
Comparing recovery efficiency across all damage types.

These results show that structural flaws can be almost entirely fixed by patching them layer by layer. On the other hand, adversarial attacks cause some performance loss that is hard to fix because they affect numerous levels. In terms of layer detection accuracy, the maximum deviation MAE based method for structural damage occasionally misattributed faults to deeper layers due to error propagation. The MAE-based maximum deviation method consistently identifies the most affected layer across scenarios.

**Table 19:** Comparison with existing repair methods.

Method	Attack type	Accuracy after repair	Repair time	Overhead
Adversarial training	FGSM	89.2	1200 s	High
NNRepair	Structural	95.1	85 s	Medium
SHNN (Proposed)	Structural + FGSM	97.3	4.2 s	Low

Quantitative comparison between SHNN and representative repair/defense methods under similar damage scenarios.

Adversarial training is expected to involve repeated attacks and full-model retrains, thus involving substantial computing resources and training time. However, methods involving constraints in repairs, such as NNRepair, involve optimization that is localized in order to modify the weights of the original networks, hence involving moderate training time. SHNN, on the other hand, freezes the original model and involves an additional lightweight patch module; in turn, repairs are significantly faster with minimal inference. Adversarial training is computationally expensive, since multiple iterations of generating attacks and full model retraining (usually over dozens of epochs and the whole dataset) are involved. NNRepair leverages localized optimization but still updates existing weights, thus it has moderate model retraining cost. By contrast, SHNN introduces a lightweight auxiliary patch (∼2.6% more parameters), while fixing the base model; this significantly reduces both repair time and inference overhead, for which quantitative results are included in Table 19.

In the current research, memory overhead implies that particular increase in memory associated with the SHNN repair technique, without taking into account the base model parameters. These include:

1. Parameters of the patch model,
2. Reference activation storage is reported separately and is not included in the patch memory overhead value.

The figure indicated in Table 5 (0.93 MB) is the memory consumption by the patch model at inference time.

Values for peak memory usage, provided in Table 20, denote total runtime memory usage (taking into account both the base model and the proposed repair model), whereas, in section “Reference activation storage overhead”, we separately evaluate the memory overhead involved in storing reference activations for localization purposes.

**Table 20:** Runtime and resource overhead of SHNN.

Metric	Base model	Patched model	Relative overhead
Parameters	246,922	253,410	+ 2.6%
Patch parameters	—	6,488	—
Patch training time (s)	—	1.7–6.5	—
Inference latency (ms / batch)	112	116	+ 3.6%
FLOPs (Forward Pass)	1.00×	1.04×	+ 4%
Peak memory (MB)	412	418	+ 1.5%

Includes total runtime memory (base model + patch module).

While Table 20 reports overall computational overhead, Table 21 focuses specifically on inference latency before and after healing.

**Table 21:** Inference efficiency before and after healing.

Model	Inference time (s)	Patch memory overhead (MB)
Original	0.144	0
Healed	0.152	0.93

Memory overhead in SHNN refers strictly to the additional parameters introduced by the patch module at inference time (reported as 0.93 MB). Runtime memory and activation storage are reported separately.

Table 21 compares inference latency before and after the insertion of the healing patch. The patched model introduces only a minor increase in inference time (approximately 0.008 s per batch), confirming that the SHNN repair mechanism maintains near real-time inference capability with negligible computational overhead.

The reported overhead values correspond to the largest patch size observed across all experiments.The inference latency was evaluated by calculating the average wall clock time for 100 forward passes with a fixed batch size of 128 for CPU inference. The patch training time is the average wall clock time taken for the auxiliary patch model learning converging, freezing the model layers as baselines. FLOPs are estimated analytically for the summation of the multiply-add operations of the Dense layers and the patch modules for a single inference pass. The maximum memory usage is acquired from the TensorFlow runtime environment for inference.

Damage localization is performed through layer-wise activation comparison.

For a batch of 128 samples, the average detection time was approximately 9–21 ms depending on batch size, indicating negligible monitoring overhead relative to inference time.

### Cross-dataset comparison

One important thing to ask about any repair system is whether it can work with more than one dataset. To further investigate this, we tested SHNN’s repair on three other domains: Numbers (handwritten digits, MNIST), Fashion (clothing items, Fashion-MNIST), and Sign (sign language digits). Table 22 summarizes the results of the experiment.

**Table 22:** Cross-dataset accuracy before damage, after damage, and after healing.

Dataset	Damage type	Pre-damage	Damaged	Healed
Numbers	Random	97.0	11.4	95.0
	Zero	97.3	9.9	94.8
	FGSM	97.7	16.0	83.0
	PGD	97.7	0.0	84.0
Fashion	Random	87.4	12.1	88.1
	Zero	86.8	9.6	86.9
	FGSM	87.7	3.0	93.0
	PGD	87.6	0.0	95.0
Sign	Random	94.1	3.8	83.5
	Zero	94.3	4.2	86.9
	FGSM	96.9	0.0	84.0
	PGD	95.3	0.0	91.0

**Dataset-level results**: SHNN is shown to be flexible and robust in repairing diverse domains with different levels of difficulty. For the domain of Numbers (MNIST), being a low-dimensional and clean domain, the accuracy of SHNN is around 98% before being damaged, and the accuracy is reduced to around 10% after being damaged with structural defects, such as random noise and zeroing, with SHNN’s repair restoring the accuracy to over 94%. The results for adversarial defects show a lower, though still good, restoration of around 83–84%.

The results for the domain of Fashion (Fashion-MNIST) show lower accuracy, around 87%, before being damaged, and the results show that SHNN’s restoration of adversarial defects (93–95%) is higher than SHNN’s restoration of structural defects (87–88%).

In the Sign Language Digits domain, which is a high-dimensional space with intricate patterns, SHNN proves its toughness in fixing issues. In such cases, accuracy drops to as low as 3–4%, but after fixing, it shoots up to 83–87%. In other challenging scenarios, where repairing FGSM and PGD adversarial defects have been applied, accuracy is restored to 84% and 91%, respectively. In scenarios where adversarial perturbations have been introduced, accuracy has been restored to as high as 83–84% for Numbers, whereas it has been restored to over 90% for Fashion and Sign datasets. Most notably, whereas PGD attacks have been harsh, their recovery has been stronger, which is attributed to SHNN’s patching, which is best at handling systematic changes.

In general, SHNN showed that it may work well in a variety of situations. It almost completely recovered in low-complexity datasets, showed better resistance to adversarial noise in moderately complex datasets, and stayed very strong in high-dimensional domains. These results show that structural faults can always be fixed, that adversarial recovery depends on the dataset, that PGD perturbations are easier to reverse than FGSM, and that SHNN is a flexible, domain-independent way to make neural networks more reliable.

#### Cross-domain patch transfer experiment

To evaluate whether SHNN’s healing mechanism generalizes beyond within-dataset repair, we conduct a cross-domain patch transfer experiment. Unlike standard cross-dataset comparisons where repair modules are retrained independently per dataset, this experiment isolates transferability by training the patch on a source domain and directly deploying it on a target domain without retraining.

Domain generalization research has shown that learned representations often encode a mixture of invariant and domain-specific features^29–31^. Methods such as invariant feature learning^29^, invariant risk minimization^30^, and distribution alignment techniques^32^ attempt to extract transferable structure across distributions. However, most prior work focuses on improving forward generalization during training rather than repairing degraded representations after deployment.

Our goal is different, however, in that we seek to test if a localized repair module is capable of transferring corrective behaviors learned under one distribution to a structurally damaged model operating under a different distribution.

**Experimental protocol**.

1. A baseline network was first trained on Dataset A.
2. Structural corruption was applied in a pre-specified layer.
3. A patch module was trained on the samples of Dataset A to correct the damaged layer.
4. The parameters of the patch module are fixed after the training.
5. The same patch module was applied to a network that was originally trained on Dataset B and had the same structural corruption.
6. No further optimization was done on the network with Dataset B.

This protocol ensures that any recovery observed on Dataset B reflects structural transfer rather than retraining adaptation.

Such a distribution shift has also been observed in other studies on adversarial robustness and neural fault tolerance, in which a model was originally trained on a given distribution and showed limited repair transfer when the feature statistics were altered^25,33^.

**Theoretical context**.

From the perspective of representation learning, the cross-domain repair is determined by the extent to which the damaged layer is able to learn domain-invariant representations. Previous studies on domain generalization showed that a deep network is able to learn a set of representations that are somewhat invariant across different domains, although the accuracy decreases when there is a distribution shift^34,35^. In addition, the theoretical study on domain adaptation showed that the accuracy of the transferred model is determined by the difference between the source and target feature distributions^31^.

If the SHNN patch has learned the activation mappings of the dataset, the cross-domain repair will not work. However, if the patch has learned a structural correction function, the cross-domain repair will work to some extent.

## Results

**Table 23:** Summarization of the results for the within-domain and cross-domain recovery between the MNIST and Fashion-MNIST datasets.

Train Domain	Test Domain	Baseline Acc (%)	Damaged Acc (%)	Healed Acc (%)	ΔAcc (%)	Recovery (%)
MNIST	MNIST	94.82 ± 0.26	8.91 ± 4.51	93.86 ± 0.96	**84.95**	**98.84**
MNIST	Fashion	9.88 ± 2.45	9.51 ± 1.28	10.05 ± 3.67	0.53	—
Fashion	Fashion	83.72 ± 1.00	10.47 ± 0.95	82.95 ± 1.02	**72.48**	**98.94**
Fashion	MNIST	9.46 ± 0.91	9.70 ± 1.06	8.50 ± 0.97	−1.20	—

Significant values are in [bold].

**Fig. 18:**
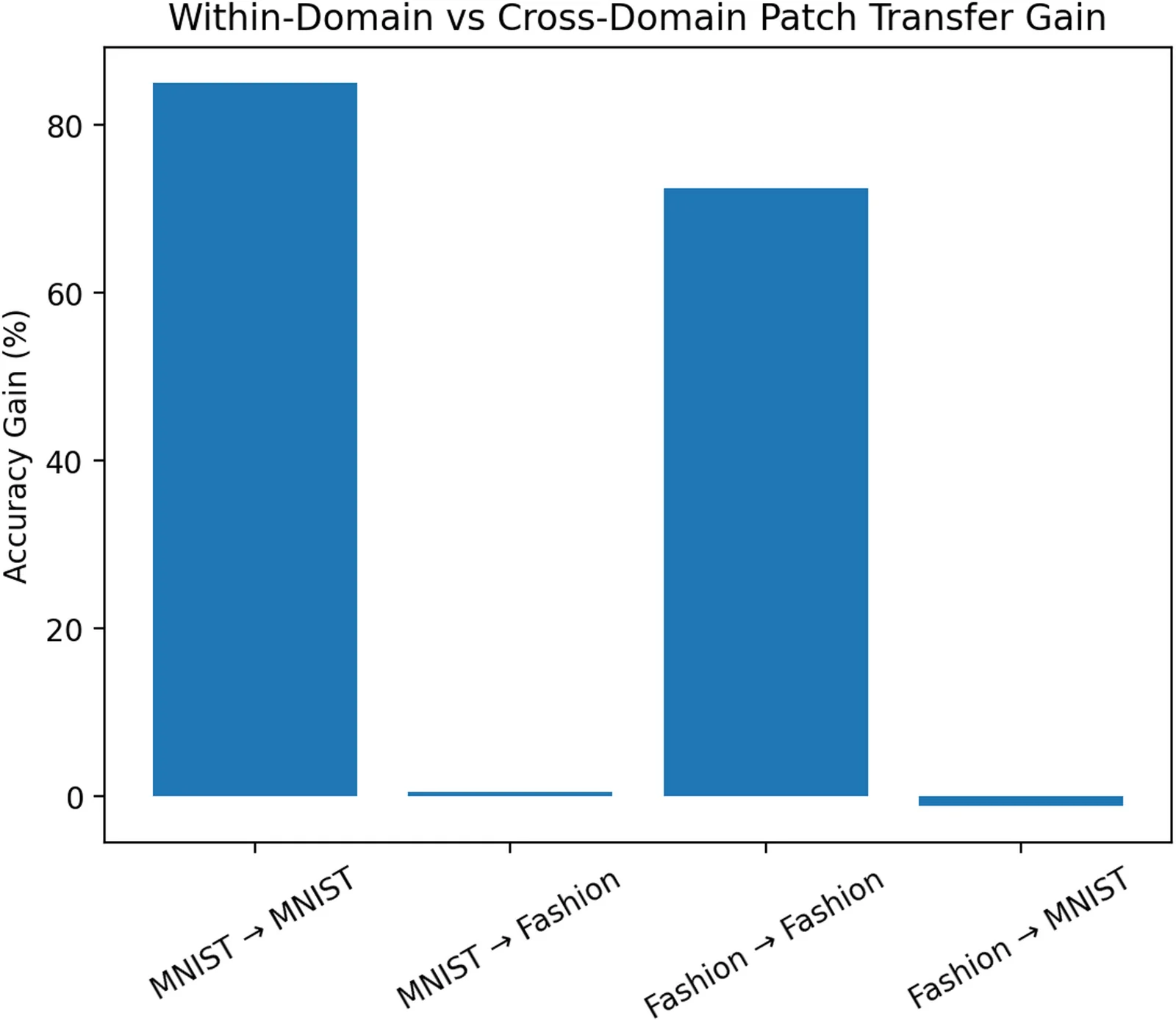
Within-domain vs. cross-domain patch transfer recovery.

## Discussion

From the results, we can see that the within-domain healing method always manages to recover the performance to near-baseline values. This shows that the patch is effectively compensating for the structural degradation, as the model is being trained and deployed in the same domain (Tables 23, 24, 25, 26, 27, 28 and 29).

In contrast, cross-domain transfer experiments show a significant drop in recovery performance. In the experiment where the patch trained on the MNIST dataset is used to recover the damaged model on the Fashion MNIST dataset, and vice versa, the accuracy of the healed model remained near the level of the damaged model, with almost no improvement over the damaged model. In some cases, even a small negative transfer is observed.

The above findings support the notion that the SHNN patch learns the domain-specific corrective mappings. Although the domain generalization literature suggests that the deep models can learn partially invariant features^29–31^, the SHNN patch seems to learn the corrective mappings that are closely related to the activation statistics of the source domain dataset.

The above findings are in accordance with the theoretical findings in the domain adaptation literature^34,35^, which show that the alignment of the representations in the two domains is not a trivial task in the absence of an invariance objective. Since the SHNN patch is not trained with the objective of domain alignment, the cross-domain transfer performance is expected to drop in the presence of a domain shift.

SHNN should thus be viewed as a localized distribution-dependent repair mechanism, with the best recovery performance obtained when the patch is trained and used in the same domain.

### Comparison with existing repair and defense methodologies

A number of approaches have been explored for improving the robustness of neural networks, as well as for repairing defective neural networks. For instance, constraint-based repair approaches, such as NNRepair, have been developed to improve the robustness of neural networks through the adjustment of network parameters based on predetermined constraints^2^. Another approach, QNNRepair, is specifically developed for the repair of quantized neural networks through the adjustment of network parameters^3^. Other approaches, such as CARE, rely on causal analysis for the repair of defective neural networks^4^.

A number of adversarial defense approaches have also been developed for the defense of neural networks against patch-based attacks. For instance, PatchZero is developed for the detection of adversarial patches, which are then removed from the input prior to the classification process^8^. On the other hand, DIFFender relies on the use of diffusion models for the suppression of adversarial attacks^9^.

In comparison, the proposed approach, namely the SHNN framework, relies on the detection of defective layers in the neural network, which are then replaced with lightweight patch modules that mimic the original transformation of the defective layer.

**Table 24:** Comparison of SHNN with existing neural repair and defense methods.

Method	Damage type	Repair strategy	Overhead	Limitation
NNRepair	Structural	Constraint optimization	High	Slow
DeepRepair	Behavioral faults	Weight modification	Medium	Retraining
PatchZero	Adversarial	Patch detection	Medium	Input-only
SHNN	Structural + adversarial	Modular patch repair	Low	Domain dependent

### Datasets used

A. Numbers (MNIST): https://www.kaggle.com/datasets/avnishnish/mnist-original/versions/1.
B. Fashion Dataset (Fashion-MNIST): https://www.kaggle.com/datasets/zalando-research/fashionmnist.
C. Sign Language (Sign Language MNIST): https://www.kaggle.com/datasets/datamunge/sign-language-mnist.
D. CIFAR-10: https://www.tensorflow.org/datasets/catalog/cifar10.

### Distributed multi-layer structural degradation

Contrary to the previously conducted experiments that test the recovery performance of the proposed SHNN model when facing single-layer corruption, in real-world deployment scenarios, there is a possibility of facing system failures in multiple internal components at the same time. For instance, structural faults, including bit-level corruption, neuron faults, or adversarial perturbations, have the potential to propagate through various layers and significantly alter the intermediate feature representations. Previous studies have demonstrated that the overall neural network inference performance is significantly impacted when facing weight-level corruption or bit-flip faults, especially when the faults propagate through the hierarchical feature representations^25^. Moreover, when facing adversarial perturbations at the initial stages of the neural network, the overall classification performance is impacted when the faults propagate through the hierarchical feature representations^23,24^.

To test the robust performance of the proposed SHNN model when facing distributed faults, we simulate the structural corruption of multiple internal components at the same time through various network layers. For each experiment, k ∈ {1, 2, 3} internal components at random are chosen and structurally damaged using the previously defined structural damage operator. Two approaches are considered for the repair mechanism: Sequential Repair, where the damaged components are repaired sequentially, and Parallel Repair, where multiple patch modules are inserted and optimized at the same time. Each experiment is conducted through multiple independent experiments using different random seeds, and the overall recovery and repair time results reported are averages over all experiments.

**Table 25:** Recovery under distributed multi-layer damage.

Damaged layers (k)	Sequential recovery (%)	Parallel recovery (%)	Sequential time (s)	Parallel time (s)
1	**98.42 ± 1.17**	**72.95 ± 25.38**	3.38 ± 0.64	3.18 ± 0.54
2	**97.80 ± 1.63**	**91.66 ± 11.29**	6.92 ± 0.96	3.70 ± 0.47
3	**97.13 ± 1.67**	**94.95 ± 4.16**	11.13 ± 1.28	4.12 ± 0.34

Significant values are in [bold].

**Table 26:** Accuracy statistics.

k	Baseline Acc (%)	Damaged Acc (%)	Seq healed Acc (%)	Par healed Acc (%)
1	97.15 ± 0.31	9.82 ± 0.19	95.94 ± 1.23	70.84 ± 24.87
2	97.15 ± 0.31	9.62 ± 0.33	94.89 ± 1.61	88.62 ± 10.85
3	97.15 ± 0.31	9.68 ± 0.29	94.04 ± 1.61	92.61 ± 3.78

The results show that the robust recovery ability of the SHNN is retained even when there is simultaneous corruption of multiple layers. Sequential repair guarantees recovery of a significant amount of the lost accuracy at all levels of corruption. Parallel repair shows greater variability when there is only one layer damaged, but its stability increases with the number of damaged layers. This shows that there is interference when there is concurrent optimization of the patch, but this interference is overcome when there is corruption of multiple layers. Similar robust recovery properties have been reported in the research on adversarial robustness and structural perturbations of deep neural networks^24,33^.

**Fig. 19:**
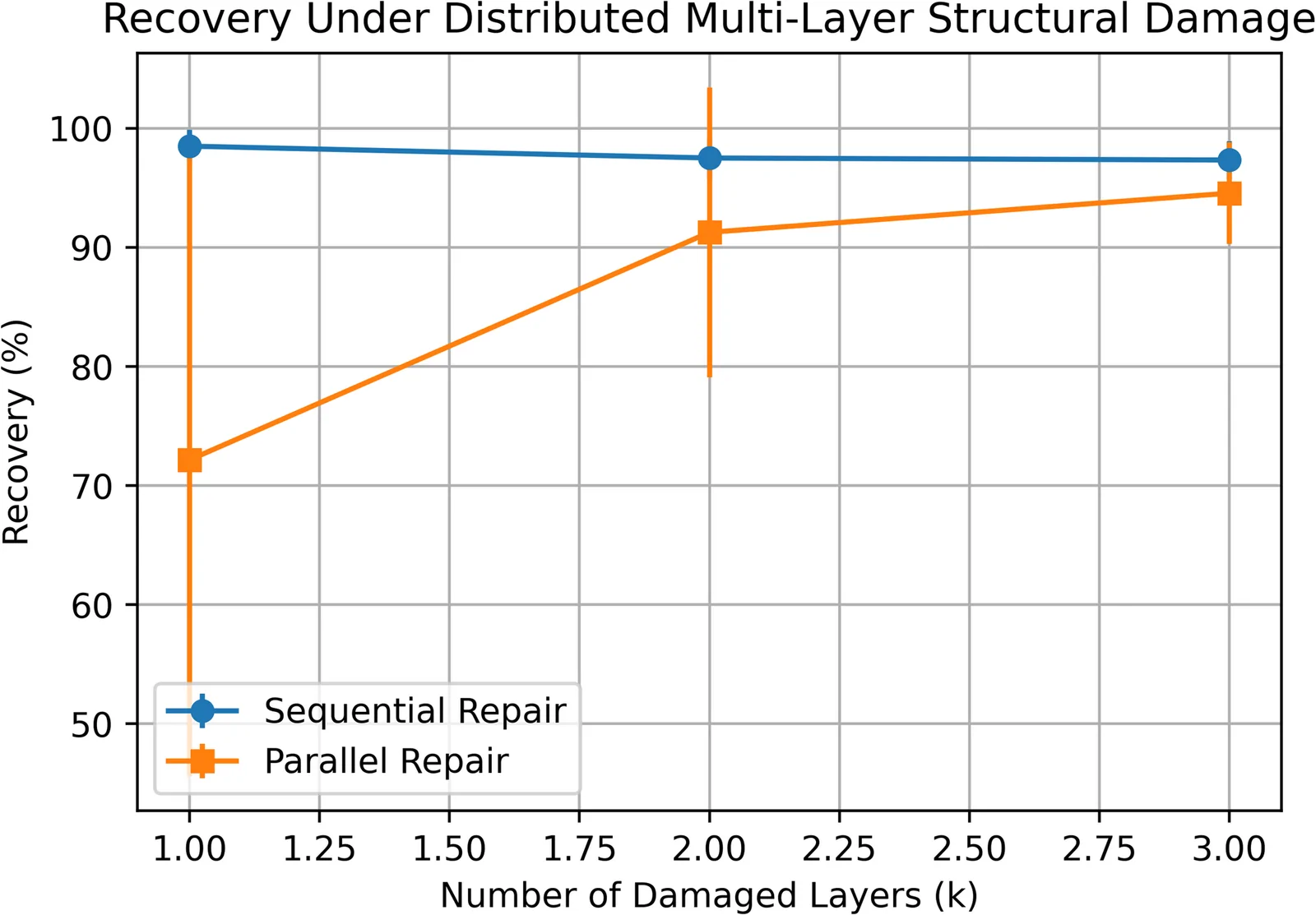
Recovery vs. number of damaged layers.

Figure 19 recovery performance of the sequential and parallel repair methods when there is distributed multi-layer structural corruption in the deep neural network.

**Fig. 20:**
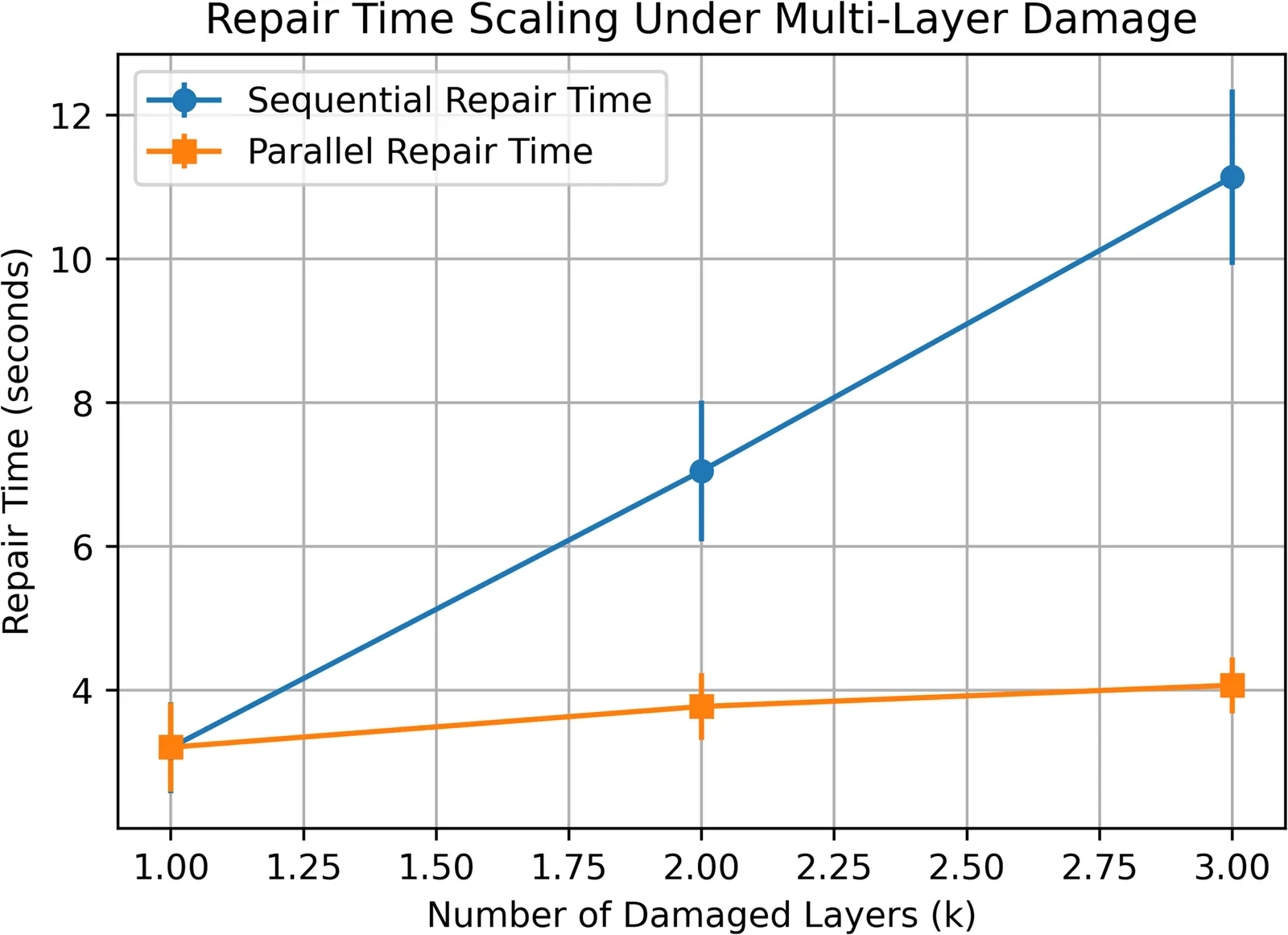
Healing time vs. damaged layer.

Figure 20 the time taken for the sequential and parallel repair methods when there is scaling with the number of simultaneously damaged layers in the deep neural network.

**Fig. 21:**
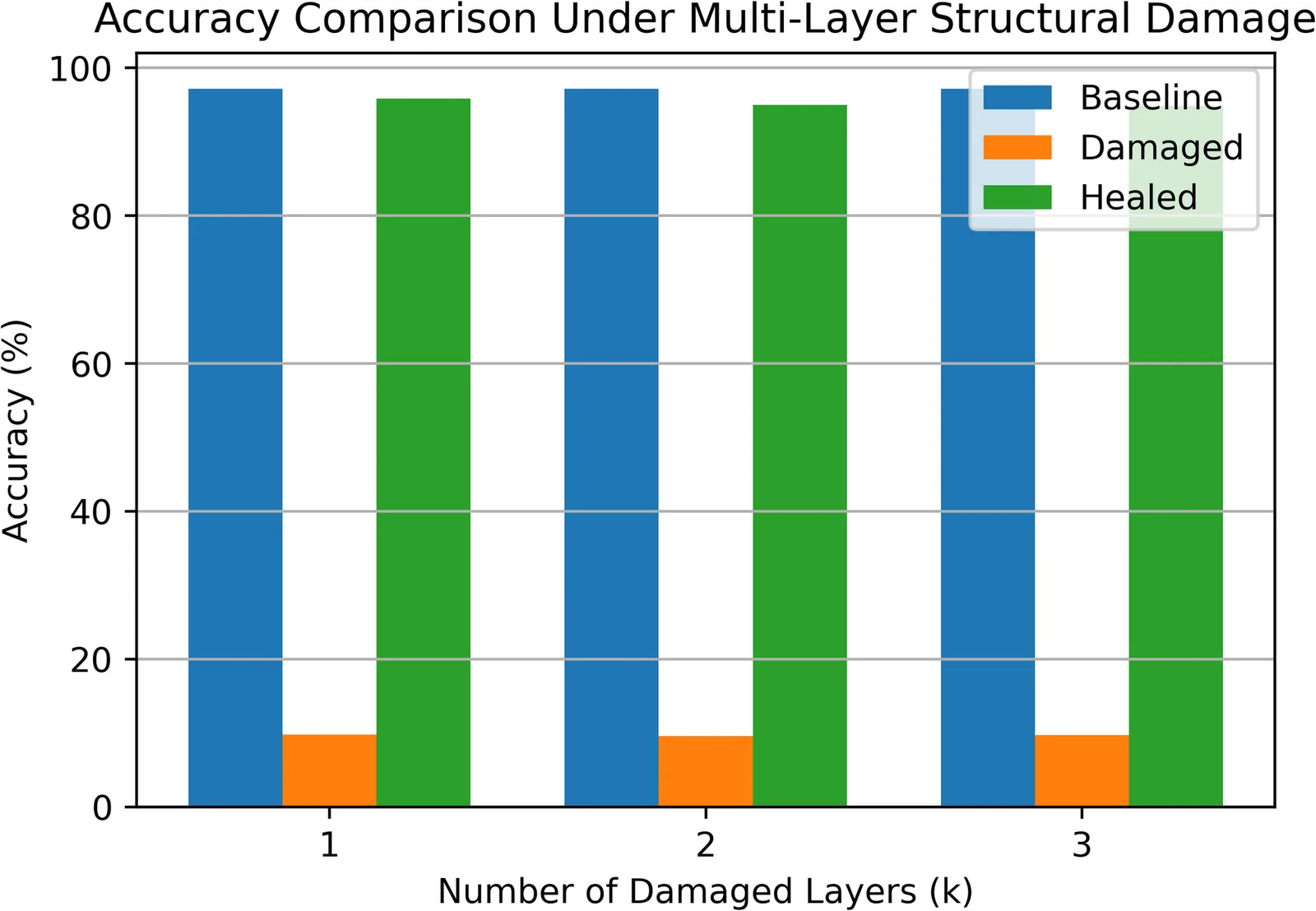
Accuracy comparison.

Figure 21 accuracy comparison for baseline, damaged, and healed states under distributed multi-layer structural corruption. As shown, the accuracy for the damaged state stays near random guessing for various values of k, since the occurrence of even one structural fault in the critical layers significantly affects the learned representation, causing the classifier to converge near chance level. Therefore, the accuracy does not drop significantly with the increase in the number of corrupted layers.

The effectiveness of the SHNN approach in recovering the accuracy under distributed corruption is also in agreement with the recent studies on the repair mechanisms of neural networks, which focus on correcting the learned representation without the need for retraining the network^36^. Therefore, the patch-based approach for correcting the internal representation of the network enables the efficient recovery of the network, even under the occurrence of multi-layer degradation.

In cases where the deviation of the activation values for the layers is almost the same, the localization mechanism selects the layer with the highest MAE deviation. As shown in the empirical results, the localization accuracy is still guaranteed under such cases, even if the deviation values for the layers are almost the same.

As an extension of the SHNN approach, the modular coordination controller can be included, which is responsible for managing the repair patches for the multi-layer degradation. This modular design would enable scalable self-healing in deeper neural architectures.

### Scalability across architectures

To verify the reliability of the SHNN model in adapting to different architectures, the SHNN model was used to detect faults in a CNN model that was originally trained on the CIFAR-10 dataset. CNN models are known to have a hierarchical structure in the feature maps obtained using convolutional filters, which makes them more susceptible to structural faults, as presented in the study on fault injection in CNNs^25^.

Structural corruption was introduced at the classifier head, reducing validation accuracy from 61.6% to 12.9%. After applying patch-based repair, performance improved to 34.9%, indicating partial recovery.

These results demonstrate that while SHNN remains functionally applicable to convolutional architectures, recovery effectiveness is reduced compared to MLPs. The distributed nature of convolutional feature hierarchies limits the degree to which single-layer patching can fully restore degraded representations.

**Table 27:** SHNN applicability across architectures.

Architecture	Validation type	Supported	Remarks
MLP	Full experiment	✔	Primary evaluation
CNN	Empirical (CIFAR-10)	✔	Classifier head repair
RNN	Conceptual	✔	Feedforward submodules
Transformer	Conceptual	✔	FFN sublayers

Although SHNN can conceptually extend to architectures containing feedforward transformation modules such as RNN projection layers or transformer feedforward blocks, empirical validation on these architectures remains future work.

**Table 28:** CNN self-healing performance on CIFAR-10.

Model state	Accuracy (%)
Baseline CNN	61.6
Damaged CNN	12.9
Healed CNN	34.9

**Fig. 22:**
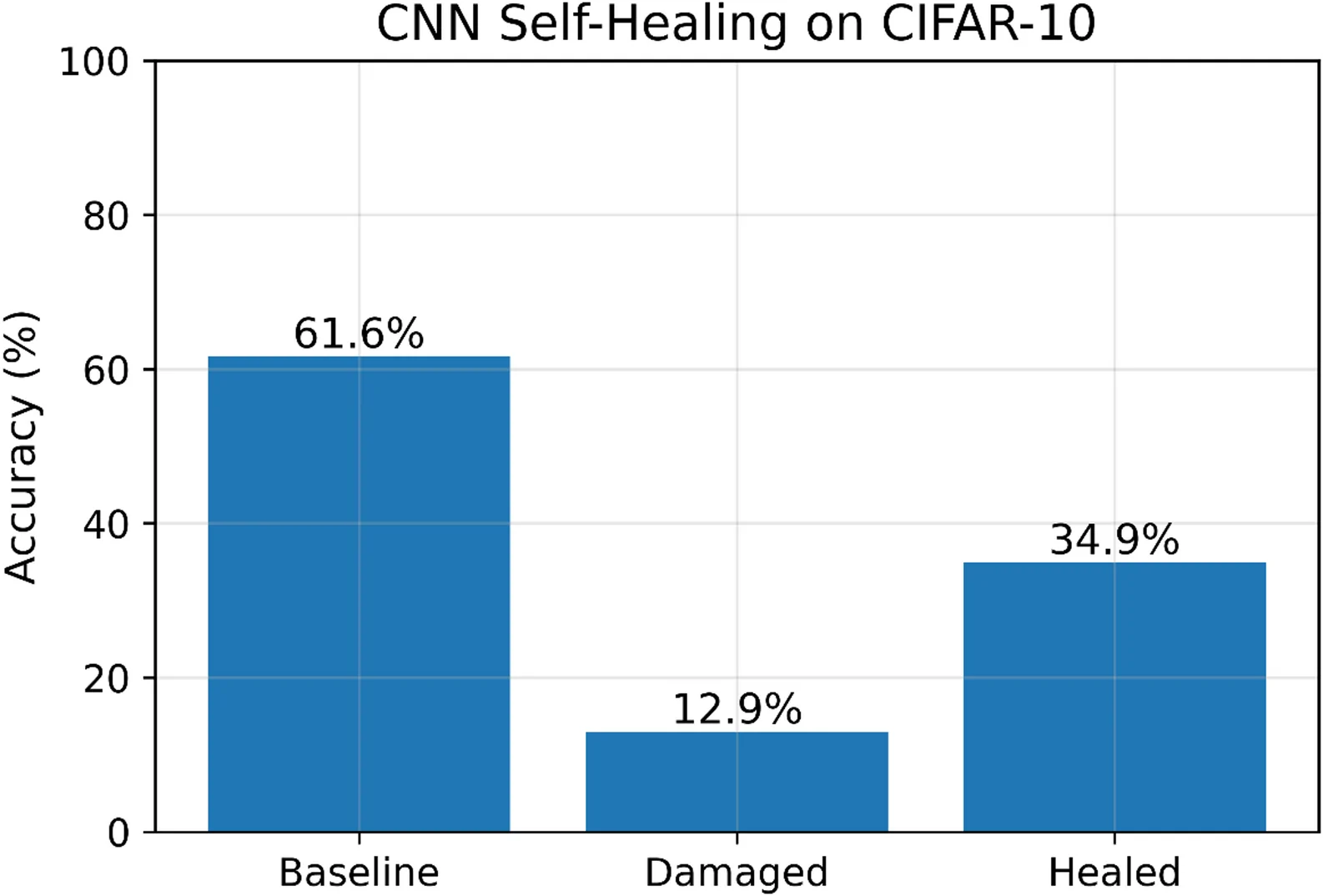
CNN baseline, damaged, and healed accuracy on CIFAR-10.

**Fig. 23:**
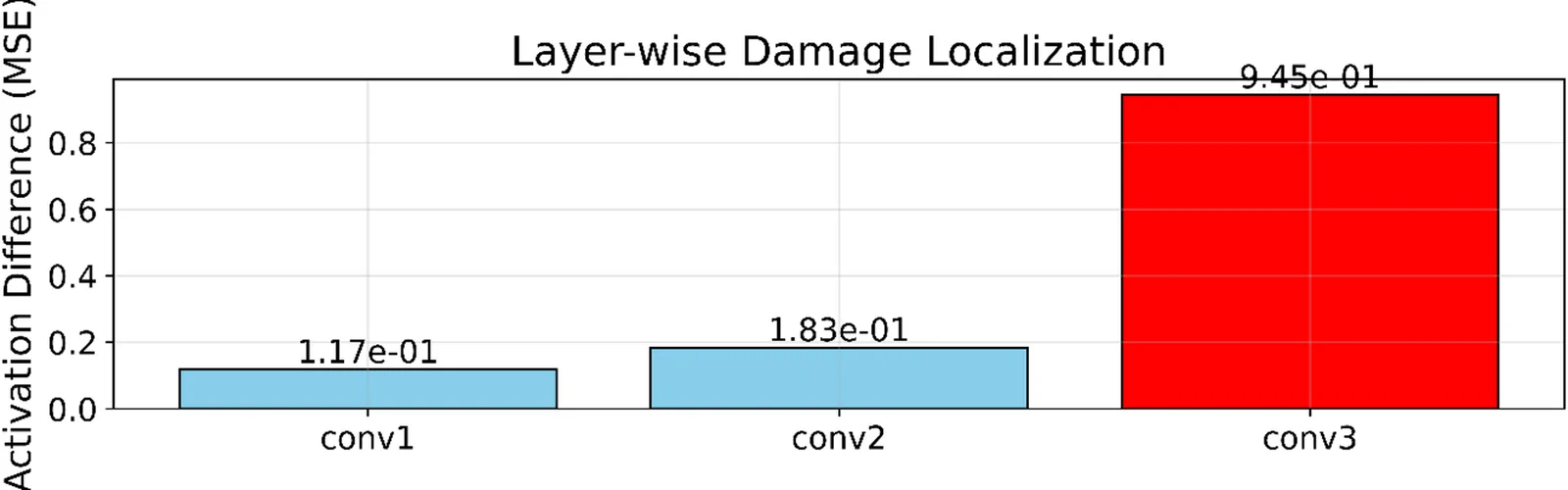
Activation deviation (MAE) used for damage localization in CNNs.

Figure 22 shows the self-healing process of CNNs on CIFAR-10. Corruption of the classifier head leads to a decrease in accuracy from 61.6% to 12.9%. However, after patch-based self-healing, the accuracy increases to 34.9%, indicating a partial and significant recovery. Unlike MLPs, CNNs spread the effects of early-layer corruption, leading to a lower ceiling of recovery (Fig. 23).

On the other hand, unlike fully connected networks, for convolutional networks, the semantic information is represented hierarchically through feature maps; hence, the error will be able to propagate through spatial feature dependencies and hence restrict the amount of recovery possible through single-layer repair. It is therefore clear that although the results obtained from the application of SHNN offer a dramatic improvement over the damaged CNN baseline, complete recovery of the baseline network is not to be expected and indicate an architectural difference rather than a limitation of the methodology.

Although preliminary experiments were conducted using a convolutional neural network on CIFAR-10, the primary focus of this study is on fully connected architectures. The proposed SHNN framework is therefore evaluated mainly on multilayer perceptron (MLP) models, which provide a controlled environment for analyzing structural damage and repair behavior. Extending the framework to deeper convolutional architectures remains an important direction for future work.

### Reference activation storage overhead

In addition to patch parameters, the SHNN framework may store reference activations for damage localization, which contributes to auxiliary memory overhead.

If N samples are stored and the activation dimension of layer l is dl, the memory requirement is:

$${\mathrm{Memory}}\, \approx \,{\mathrm{N}} \times {\mathrm{dl}} \times {\mathrm{sizeof}}\left( {{\mathrm{float}}} \right)$$

For MNIST experiments, storing activations for 10,000 samples at the dense2 layer (64 units) requires:

$${\mathrm{1}}0,000{\text{ }} \times {\text{ 64 }} \times {\text{ 4 bytes}}\, \approx \,{\mathrm{2}}.{\text{56 MB}}.$$

The low memory requirement is also useful for activation monitoring, particularly for edge devices with low resources.

**Table 29:** Memory overhead of activation storage.

Layer	Activation size	Samples	Memory
dense0	256	10k	10 MB
dense1	128	10k	5 MB
dense2	64	10k	2.56 MB

Higher clean accuracies observed in adversarial tables arise due to evaluation on a smaller fixed subset and are not directly comparable to full test set accuracy.

## Discussion and implications

The experimental study of the Self-Healing Neural Network (SHNN) structure indicates its potential for mitigating both adversarial and structural damages, as well as the factors affecting the detection and healing efficiency of the network. The following section will discuss the results, conclude, and interpret the results in the context of neural network robustness research.

### A point of reference: the strength of the baseline model

The baseline network, having been pretrained using MNIST and trained for only 10 epochs, achieved a training accuracy of 99.43% and a test accuracy of 97.76%, an extremely high value for such a short training period. This confirms the suitability of using moderately trained networks as a candidate for network damage healing research, as they are still quite effective despite being poorly trained. The network is also relatively small, with three hidden layers and using ReLU as the activation function, and a softmax layer for the output, making it easier to assess the extent of the network damage and healing, as the changes in network performance will be directly attributable to the network damage or healing and will not be due to the network’s inability to perform the task, as is the case with other networks. Additionally, the network’s structure allows for accurate identification of the damaged layer using metrics such as MAE(used for localization), as each layer is responsible for a particular aspect of feature abstraction, and the extent of the damage can be pinpointed with relative precision.

### Effect of group destructive harm

FGSM Attack: The FGSM attack, with ε = 0.2, resulted in a loss of network accuracy, dropping from 98% for clean inputs to only 10% for adversarial inputs, indicating the network’s vulnerability to gradient-based attacks, which exploit weaknesses in decision boundaries, as is the case with feedforward networks. Although the FGSM is a single-step attack, it still managed to corrupt the mid-to-late stage features, with dense2 recording the highest MSE of 4.4.

The PGD assault (ε = 0.20, α = 0.01, 30 iterations) was significantly worse.

PGD Attack: The PGD attack (ε = 0.20, α = 0.01, 30 iterations) was even more severe. Accuracy fell from 100% for clean inputs to 0% for the adversarial examples. This rendered the classifier useless for the classification task. The dense2 layer was the most affected, with the highest mean squared error (MSE) of 10, which was more than double the value of the MSE in the FGSM scenario. This concurs with the understanding of PGD attacks, which amplify the strength of the attacks and focus on the most sensitive features of the model in each iteration. The fact that the dense2 layer was affected in both scenarios indicates the layer’s vulnerability profile, which could be useful in developing proactive mitigation techniques such as regularization, adversarial training, and layer redundancies for the key mid-to-late layer in the model.

### Efficiency of cure adversarial recovery

In the adversarial scenario, the SHNN’s patch-based recovery mechanism was seen to perform well. After healing, the accuracy of the model for the hostile examples was 84% using the FGSM scenario, while the accuracy for the clean examples was 98%. This is an improvement of nearly 74%. Although the attack was severe in the beginning, the PGD recovery mechanism was seen to achieve 84% accuracy for the adversarial examples and 95% for the clean examples. This indicates the effectiveness of the recovery mechanism. What is more interesting is the fact that the recovery rate for the PGD scenario was quite close to the recovery rate for the FGSM scenario, even though the PGSM scenario’s pre-healing accuracy was 0%. This indicates the effectiveness of the patch replacement mechanism in the recovery scenario. However, the fact that the FGSM scenario had 16% error rate for the adversarial examples and the PGSM scenario had the same error rate even after healing indicates that the healing mechanism was effective in repairing the feature representations but was not effective in reconstituting the robustness of the decision boundary.

### A view on structural damage

When the hidden layer is set to zero, the network is unable to effectively represent the information, leading to a significant decline in accuracy from 97.76% to 9.94%. In this case, the damage-detection module was able to effectively identify the main causes of the failure, with dense1 being the main contributor to the failure, despite the fact that the MAE values were higher for dense2 due to the propagated faults. This shows the significance of threshold-based techniques, whereby the top-MAE method is not sufficient for the identification of the causes of the failure in the neural network. After the application of the proposed self-healing patch, the accuracy of the model is restored to 97.33%, showing almost full recovery from the damages inflicted, with 87.39% of the lost performance being regained. When the layer is replaced with zero, the accuracy of the model is significantly reduced to 10.33%, although this is slightly less severe than the zeroing case. After the application of the proposed self-healing patch, the accuracy is restored to 97.22%, showing almost full recovery from the damages inflicted, with 87% of the lost performance being regained.

### Comparison recovery trend

Table 7 shows a comparative study that helps clarify some important aspects. To begin with, it has been seen that accuracy loss resulting from structural damage can always be made up for, with a post-healing accuracy within 0.5% of the baseline. This shows how effective SHNN’s patch-based repair mechanism is in remedying structural damage confined to a single region. Secondly, it has been noted that adversarial damage is harder to make up for, possibly because this type of damage involves changes to multiple layers simultaneously, altering the decision boundaries in a way that cannot easily be corrected by a single-layer remedy. Thirdly, it has been seen that the percentage of recovery from PGD attacks was higher than that from FGSM, even though PGD has a much larger initial impact. This shows that PGD attacks tend to concentrate in particular layers, such as dense2, making it easier to isolate and correct, while FGSM has a more diffuse, coarse-grained effect.

### MAE/MSE as metrics for damage determination metrics

The results of the empirical analysis suggest that the effectiveness of damage detection at the layer level depends on the type of fault that occurs. For instance, the MAE threshold approach was the most effective for structural damage detection due to its capacity to identify changes that first occur in the first layer. On the other hand, the peak MAE approach was the most effective for adversarial damage detection, as malicious changes occur across several layers. As such, the layer that experiences the maximum MAE value is the most affected. As such, the results suggest that the type of damage that occurs in the network determines the approach that is used for detection. As such, the results suggest that the type of damage that occurs in the network determines the approach that is used for detection. As such, the results suggest the need for the development of adaptive approaches for network damage detection, as none of the metrics can effectively detect all types of network deterioration that occur due to various types of network failures.

### Broader consequences and limitations

The results of the analysis have also made an important contribution to the existing body of knowledge on the development of self-repairing neural networks, as they suggest that the patching of layers is an alternative approach to the retraining of the entire network, particularly for mission-critical systems that require immediate repair. For instance, the results suggest that the network can recover almost full functionality with little data and computational requirements in the presence of structural damage. However, the results also suggest that the network is still vulnerable to adversarial attacks, which is a major limitation. As such, the results suggest that the patching approach does not improve the network’s capacity to recover from adversarial attacks. As such, the results suggest that the patching approach does not improve the network’s capacity to recover from adversarial attacks. As such, the results suggest the need for the use of the patching approach in combination with other approaches, such as gradient masking. Another limitation of the results is that they were based on the MNIST dataset using the simplest network architecture. We don’t know whether the SHNN method will work with deeper networks and more complex datasets (such as CIFAR-10 and ImageNet), because deeper networks might exhibit more dispersed damage patterns, especially when there are significant structural faults.

Although this work examines FGSM and PGD attacks, more sophisticated multi-layer adversarial attacks like DAG and AIR were not directly implemented in this work. This is because these attacks involve the distribution of perturbations over multiple layers, which necessitates multi-patch coordination, an area we propose for future research. The reduced recovery observed in convolutional networks highlights that SHNN’s single-layer patch strategy is most effective when degradation is localized and weakly entangled, and may require multi-layer coordination in deeper hierarchical models.

### Cross-dataset generalization

Cross-dataset experiments show that SHNN is not restricted to a single domain. Whether applied to digit recognition (Numbers), fashion classification, or sign language gestures, the same repair model recovered most of the lost performance. The current results show that, instead, the healing mechanism is dynamic and learns to adapt to the structural characteristics of neural representations. Another important finding is that, despite their differences, all datasets showed high structural recovery, with nearly complete restoration, even after extensive damage. In contrast, adversarial recovery is less consistent and is dataset-dependent, especially concerning dataset complexity. For instance, the Fashion-MNIST dataset showed a high recovery rate under the PGD attack, 95%, whereas the Sign dataset plateaued at 91%. This indicates that SHNN’s generalization is robust but still dependent on the level of feature dissimilarity and how well features can be used to differentiate between classes. These findings, therefore, provide important insights into SHNN’s healing capability, both in terms of its ability to heal localized structural damage and its limitations regarding adversarial attacks, which is important for developing SHNN as a holistic approach to neural network self-repair.

### Reference model maintenance and threshold sensitivity

The SHNN healing mechanism currently uses predefined activation functions and thresholds for MAE/MSE. In systems that have been running for a long time, it is possible to have reference drift. This is where drift-aware mechanisms, such as recalibrating clean activation baselines at regular intervals, might be useful. In terms of sensitivity, it has been observed that lower threshold values improve detection times but increase false positives, whereas higher threshold values improve false positive reduction but increase false negatives. These are areas that might be explored further. In terms of current findings, it has been observed that, empirically, MAE threshold values ranging between 0.08 and 0.12 provide the best compromise between false positives and false negatives.

### Failure modes and limitations

Although the proposed Self-Healing Neural Network (SHNN) framework demonstrates strong recovery characteristics for both structural corruption cases and adversarial attacks, some limitations need to be considered for the implementation of the repair approaches.

#### Limited effectiveness for distributed damage cases

The proposed repair approach for the SHNN, based on the detection of the dominating corrupted layer through activation deviation analysis, may not always show optimal results. In cases where the network is corrupted at several layers simultaneously, the patch module may fail to approximate the original transformation function of the corrupted layer. As mentioned earlier, the repair approach for the neural network, based on the detection of the corrupted layer, may fail for cases of distributed damage^37^.

#### Reduced recovery for strong adversarial attacks

Empirical results reveal that patch-based repair techniques achieve near-full accuracy after structural corruption, compared to the partial recovery of the model under adversarial attacks. For example, PGD attacks alter the internal activations of the model across various layers, making the repair process more challenging. As discussed in the literature for adversarial robustness, the stronger the iterative attacks, the larger the space of possible attacks, which may necessitate the use of model-wide adversarial training^38,39^.

#### Dependence on accurate damage localization

The efficacy of the SHNN repair mechanism relies on the precise detection of the corrupted layer using the activation deviation metrics. However, in cases where multiple layers exhibit similar activation deviations, the layer localization mechanism may incorrectly identify the deeper layer affected by the propagation of errors rather than the original layer of corruption. Automated neural network testing tools have previously reported difficulties in identifying the root cause of failure in deep neural networks^40^.

#### Architectural scalability constraints

Although the proposed SHNN repair mechanism is designed for feedforward neural networks, the repair mechanism may struggle to handle the complex feature hierarchies and spatial correlations in deep convolutional networks. Previous studies on neural network compression and redundancy have reported the presence of additional dependencies in deeper neural networks, which may make the repair mechanism less effective^41^.

#### Potential risk of overfitting in the patch modules

Though the patch network is kept simple, there is a potential risk of overfitting in the repair data, especially due to the presence of corrupted activations in the network. Moreover, the risk is further pronounced in the presence of divergent damage patterns in the network. Domain generalization studies have proved that the corrective mappings are not always effective in the presence of different statistical distributions in the data sets^28^.

Despite the limitations and the risks, the SHNN framework is a viable solution to the problem of repairing the neural network, and it is computationally efficient. In the future, the SHNN framework could be extended to include the coordination of the multi-patch network, the localization metrics, and the repair strategies in the context of the deep convolutional network.

## Conclusions

In this paper, the Self-Healing Neural Network (SHNN) architecture is proposed, and it is a system that is capable of identifying and repairing the problems in the higher layers of the network, which are caused due to structural faults or due to the presence of adversarial faults targeting the higher layers. Employing the miniature “patch” network training and applying it exactly where the damage is incurred, SHNN was able to repair the network to almost full functionality after structural damage, recovering significantly from the loss of accuracy to adversarial noise, without the cost and time of retraining an entire model. Our data indicate that, in the localized-damage case, a well-positioned patch is quite effective. The same technique also aids in adversarial cases, although in these cases the recovery is modester — implying that mending works best when the damage is focal, and various approaches are required when attacks transmit their effects via a series of layers.

Since SHNN offers modularity and lightness, it can readily be applied in practice systems that prioritize recovery velocity. The GUI we developed as an interaction facilitates real-time observation, detection, and repair of models, converting what could have been a labor-intensive maintenance procedure into a fast, targeted one. For the purpose of future applications, the SHNN framework could be extended to accommodate the higher-level data and the higher number of patches working in concert. With the help of future advancements in the training algorithms, the system could potentially lead to the creation of a self-aware system that is capable of self-repair in the presence of faults in the network.

## Limitations and future work

Firstly, the multi-layer patching could be used to patch the network across several layers, which would help in addressing the problems caused due to the presence of adversarial faults, which affect the network across several layers. Secondly, the patch network could be adapted using the adversarial examples, which would help in increasing the robustness of the network. Thirdly, the addition of the parallel paths to the sensitive levels, such as the dense2 level, would help in increasing the redundancy in the network and would also help in addressing the problem of single-point failures in the network. Finally, the hybrid detection metrics, which combine the MAE thresholding and the real-time peak MAE, could also help in increasing the robustness of the network. By integrating these refinements, SHNN can evolve into a globally stable self-repair framework capable of addressing both localized structural faults and distributed adversarial degradations with greater efficiency and precision.

## Data Availability

Data availabilityMNIST - The datasets generated and/or analysed during the current study are available in the [MNIST] repository, https://www.tensorflow.org/datasets/catalog/mnistFashion-MNIST — The datasets generated and/or analysed during the current study are available in the [Zalando Research Fashion-MNIST] repository, https://github.com/zalandoresearch/fashion-mnistSign Language MNIST — The datasets generated and/or analysed during the current study are available in the [Kaggle Sign Language MNIST] repository, https://www.kaggle.com/datasets/datamunge/sign-language-mnistCIFAR-10 — The datasets generated and/or analysed during the current study are available in the [CIFAR-10 / Alex Krizhevsky] repository, https://www.cs.toronto.edu/~kriz/cifar.html.

## Code availability

The custom implementation developed in support of this research project, including the SHNN framework, structural damage simulation routines, adversarial attacks creation (FGSM and PGD), activation difference detection, modular patch-based healing approaches, statistical assessment pipelines, and Streamlit-based visual interface, were implemented in Python utilizing TensorFlow/Keras. The source code and implementation details necessary to reproduce the results presented in this paper are available upon reasonable request from the corresponding author. The implementation includes: Base MLP and CNN models, Structural damage simulation routines, Adversarial attacks generation, Activation localization algorithms, Healing patches training pipelines, Statistical assessment and ablation studies, Streamlit visual interface.
